# Predicting the tumor microenvironment composition and immunotherapy response in non-small cell lung cancer from digital histopathology images

**DOI:** 10.1038/s41698-024-00765-w

**Published:** 2024-12-19

**Authors:** Sushant Patkar, Alex Chen, Alina Basnet, Amber Bixby, Rahul Rajendran, Rachel Chernet, Susan Faso, Prashanth Ashok Kumar, Devashish Desai, Ola El-Zammar, Christopher Curtiss, Saverio J. Carello, Michel R. Nasr, Peter Choyke, Stephanie Harmon, Baris Turkbey, Tamara Jamaspishvili

**Affiliations:** 1 Artificial Intelligence Resource (AIR), National Cancer Institute, National Institutes of Health, Bethesda, MD USA; 2 Department of Hematology and Oncology, SUNY Upstate Medical University, Syracuse, NY USA; 3 Department of Pathology and Laboratory Medicine, SUNY Upstate Medical University, Syracuse, NY USA

**Keywords:** Cancer imaging, Cancer microenvironment, Non-small-cell lung cancer, Tumour biomarkers

## Abstract

Immune checkpoint inhibitors (ICI) have become integral to treatment of non-small cell lung cancer (NSCLC). However, reliable biomarkers predictive of immunotherapy efficacy are limited. Here, we introduce HistoTME, a novel weakly supervised deep learning approach to infer the tumor microenvironment (TME) composition directly from histopathology images of NSCLC patients. We show that HistoTME accurately predicts the expression of 30 distinct cell type-specific molecular signatures directly from whole slide images, achieving an average Pearson correlation of 0.5 with the ground truth on independent tumor cohorts. Furthermore, we find that HistoTME-predicted microenvironment signatures and their underlying interactions improve prognostication of lung cancer patients receiving immunotherapy, achieving an AUROC of 0.75 [95% CI: 0.61-0.88] for predicting treatment responses following first-line ICI treatment, utilizing an external clinical cohort of 652 patients. Collectively, HistoTME presents an effective approach for interrogating the TME and predicting ICI response, complementing PD-L1 expression, and bringing us closer to personalized immuno-oncology.

## Introduction

Lung cancer is the leading cause of cancer-related mortality globally, of which non-small cell lung cancer (NSCLC) is the most common histological subtype^1^. In recent years, immune checkpoint inhibitors (ICI) have radically transformed the prognosis of clinically advanced NSCLC. Although ICI-treatment is approved for the treatment of NSCLC patients in the localized and metastatic setting regardless of PD-L1 status^2–4^, many patients still fail to achieve clinically meaningful responses. Considering the high costs of treatment and potential immune related toxicities^5,6^, identifying biomarkers of anti-PD1/PDL1 treatment response is of critical importance. The use of PD-L1 as a biomarker to determine which patients respond to ICI-treatment is hindered by challenges associated with standardized quantification of PD-L1 expression^7^. Controversies such as responders with low PD-L1 expression levels and non-responders with high PD-L1 levels are very common^8^. For example, after selecting patients based on PD-L1 expression, response rates to treatment still vary widely, ranging from 17 – 49% in patients with Tumor Proportion Score (TPS) > 1%^9^. These discrepancies could be attributed to a high level of intratumor heterogeneity of PDL1 expression and the underlying complexity of tumor microenvironment^10^. The clinical utility of other biomarkers such as the Tumor Mutational Burden (TMB) has been explored in several different clinical trials^11–14^. However, both PDL-1 expression and TMB fail to encapsulate various tumor microenvironmental features influencing ICI responses^15–17^. Hence, there is a clinically unmet need for additional predictive biomarkers capturing both tumor and microenvironmental factors associated with ICI responses.

In recent years, several new multiplex tissue imaging and spatial transcriptomics technologies have been used for profiling the tumor microenvironment (TME) of patients in unprecedented detail^18–26^. However, they are quite expensive, which does not allow them to be implemented for wider use in a clinical setting. Hematoxylin and Eosin (H&E)-stained pathology slides, on the other hand, are relatively cheap and easily accessible in any pathology lab. These slides hold a wealth of TME-related information that can be unlocked with the help of Artificial Intelligence (AI)^27^. One of the first large-scale attempts to characterize the TME of patients from H&E slides was the work of Saltz et al., who mapped the abundance and spatial distribution of tumor infiltrating lymphocytes (TILs) across 23 different cancer types^28,29^. Graham et al. developed Hover-Net^30^, a pan-cancer nuclei segmentation and classification neural network that enables single-cell quantification of tumor, stroma, and lymphocyte populations from H&E slides. More recently, Diao et al. developed a collection of supervised machine learning (ML) methods to quantify 607 human interpretable TME features from histopathology images^31^. While these approaches are extremely valuable, they are limited by availability of relevant pixel-level annotations from expert pathologists, which are time and resource consuming to generate. To overcome these limitations, several research groups have alternatively proposed the use of weakly-supervised deep learning models, which can be trained to perform various downstream computational pathology tasks such as tumor subtyping and prognosis^32–37^ without any region or pixel-level annotations.

Building on these recent AI-based advances, we introduce HistoTME: a weakly supervised multi-task learning approach to infer the TME composition of patients from routinely collected pathology slides. Unlike previous approaches, HistoTME harnesses recently developed digital pathology foundation models to infer the expression of 30 distinct cell type-specific TME signatures, previously shown to be associated with immunotherapy responses^38^. HistoTME is trained in a weakly supervised fashion utilizing matched whole slide H&E and bulk transcriptomics data of 865 NSCLC patients from The Cancer Genome Atlas (TCGA), validated on matched whole slide H&E and bulk transcriptomic data of 333 NSCLC patients from the Clinical Proteomic Tumor Analysis Consortium (CPTAC) and tested on whole slide H&E and IHC data from 82 NSCLC patients that had complete surgical resection at SUNY Upstate Medical University. We further demonstrate the clinical utility of HistoTME predictions by retrospectively analyzing needle biopsy specimens and clinical outcome data from an additional 570 NSCLC patients from the SUNY Upstate cohort treated with either chemotherapy or immune checkpoint inhibitors. Importantly, we show that HistoTME AI scores complement low PD-L1 expression and can identify more patients responding to first line immune checkpoint inhibitor therapy. Taken together, HistoTME presents a versatile and accessible tool for unraveling the complex dynamics of TME, leading to improved risk stratification and management of NSCLC patients.

## Results

### Overview of HistoTME

**Fig. 1:**
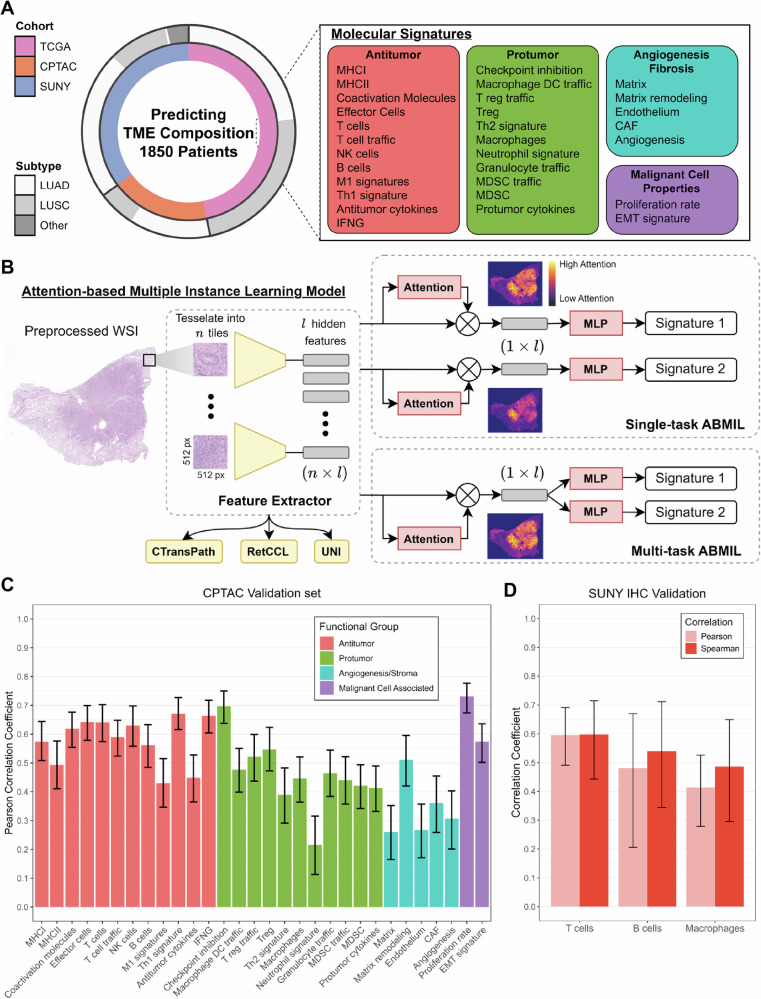
Overview of pipeline and performance of HistoTME. **A**, **B** Each Whole Slide Image is tessellated into smaller tiles and preprocessed by a pretrained digital pathology foundation model to extract meaningful tile embeddings. The tile embeddings generated by the foundation model are then provided as input to an attention-based multiple instance learning (AB-MIL) module followed by a multi-layer perceptron head (MLP), which learns to predict expression levels to 30 tumor microenvironment-related molecular signatures. Overall, to develop HistoTME we experiment with three open-source foundation models—CTransPath, RetCCL, and UNI^21,22,26^—and two configurations of AB-MIL: single task AB-MIL, where the predictions of each signature are optimized separately, and multi-task AB-MIL, where predictions of functionally related signatures are jointly optimized. The signature prediction performance of each foundation model coupled with each configuration of AB-MIL is shown on held out CPTAC validation data in Supplementary Fig. 3. Overall, the UNI foundation model + multitask AB-MIL produces the most accurate predictions and is hence chosen as the final version of HistoTME. **C** Pearson correlations between the ground truth expression levels of each patient derived from bulk transcriptomics and predicted expression levels of each patient derived from the final version of HistoTME (UNI+multi-task AB-MIL) on the held out CPTAC validation cohort. **D** Pearson and Spearman correlations between the cell type abundance of each patient, defined as the number of marker positive cells per mm^2^ from immunohistochemistry (IHC) slides, and the predicted cell type-specific signature expression levels of each patient derived from final version of HistoTME (UNI+multitask AB-MIL) is shown on the external SUNY Upstate test cohort. Error bars represent the 95% confidence intervals. Cell type abundances were estimated from whole slide immunohistochemistry images using QuPath v0.5.0 cell detection and classification algorithms with default parameter settings. *TME* tumor microenvironment, *LUAD* lung adenocarcinoma, *LUSC* lung squamous cell carcinoma, *MLP* multilayer perceptron.

HistoTME is a deep learning model trained to predict the average normalized gene expression levels of 30 cell type-specific TME signatures from whole slide H&E images, collectively providing a comprehensive profile of the TME composition^38,39^ (Fig. 1A). HistoTME was trained using whole slide images (WSI) of H&E staining and matched bulk transcriptomics data from the TCGA-NSCLC cohort (*N* = 865 patients) and validated using an external cohort of 333 NSCLC patients from CPTAC using the same data modalities (Supplementary Fig. 1). The HistoTME model consists of two main components: a frozen feature extraction component and a trainable attention-based multiple instance learning (AB-MIL) component^40^. In our efforts to efficiently train HistoTME, we explored three state-of-the-art open-source foundational models—CTransPath, RetCCL, and UNI^32,33,37^—as potential feature extractors. Additionally, we conducted experiments with two distinct approaches for AB-MIL: a single-task approach featuring a unique attention and multilayer perceptron (MLP) head for each TME signature, and a multitask approach, which incorporates a shared attention head for functionally related TME signatures but maintains separate MLP heads for each individual TME signature (Fig. 1B).

### HistoTME accurately infers the TME composition of NSCLC patients from histopathology images

Of all versions of HistoTME that were explored, we observed that the version utilizing multi-task AB-MIL + the UNI foundation model produced the most accurate predictions when compared with the ground truth, achieving an average Pearson correlation coefficient of 0.50 (Fig. 1C, Supplementary Fig. 2A). The performance of single-task AB-MIL + UNI was slightly worse than the performance of multi-task AB-MIL + UNI. However, both single-task AB-MIL + UNI and multi-task AB-MIL + UNI significantly outperformed other versions of HistoTME for predicting antitumor and protumor immune signatures while displaying similar performance for other signature prediction tasks (Supplementary Fig. 3). Therefore, we settled on multitask AB-MIL + UNI as the final version of HistoTME. Having validated the accuracy of HistoTME on CPTAC-NSCLC data, we next tested HistoTME on whole slide H&E images of 82 NSCLC patients from SUNY Upstate Medical University, which had serial immunohistochemistry (IHC) performed on surgical resection specimens for immune cell panel: T cells (CD3, CD4, DC8), B cells (CD20) and Macrophage (CD163) markers using adjacent serial sections (Supplementary Fig. 1). Overall, we found that HistoTME predicted expression levels were correlated with the abundance of each cell type derived from IHC, achieving Pearson correlations of 0.60 [95% CI: 0.49-0.69] for T cells, 0.48 [95% CI: 0.21-0.67] for B cells, and 0.41 [95% CI: 0.28-0.53] for macrophages, and Spearman correlations of 0.60 [95% CI: 0.44-0.72] for T cells, 0.54 [95% CI: 0.34-0.71] for B cells, and 0.49 [95% CI: 0.30-0.65] for macrophages (Fig. 1D, Supplementary Fig. 2B). Spearman correlations were included due to the uncertain linear correlation between gene expression and IHC-measured protein abundance^41^.

**Fig. 2:**
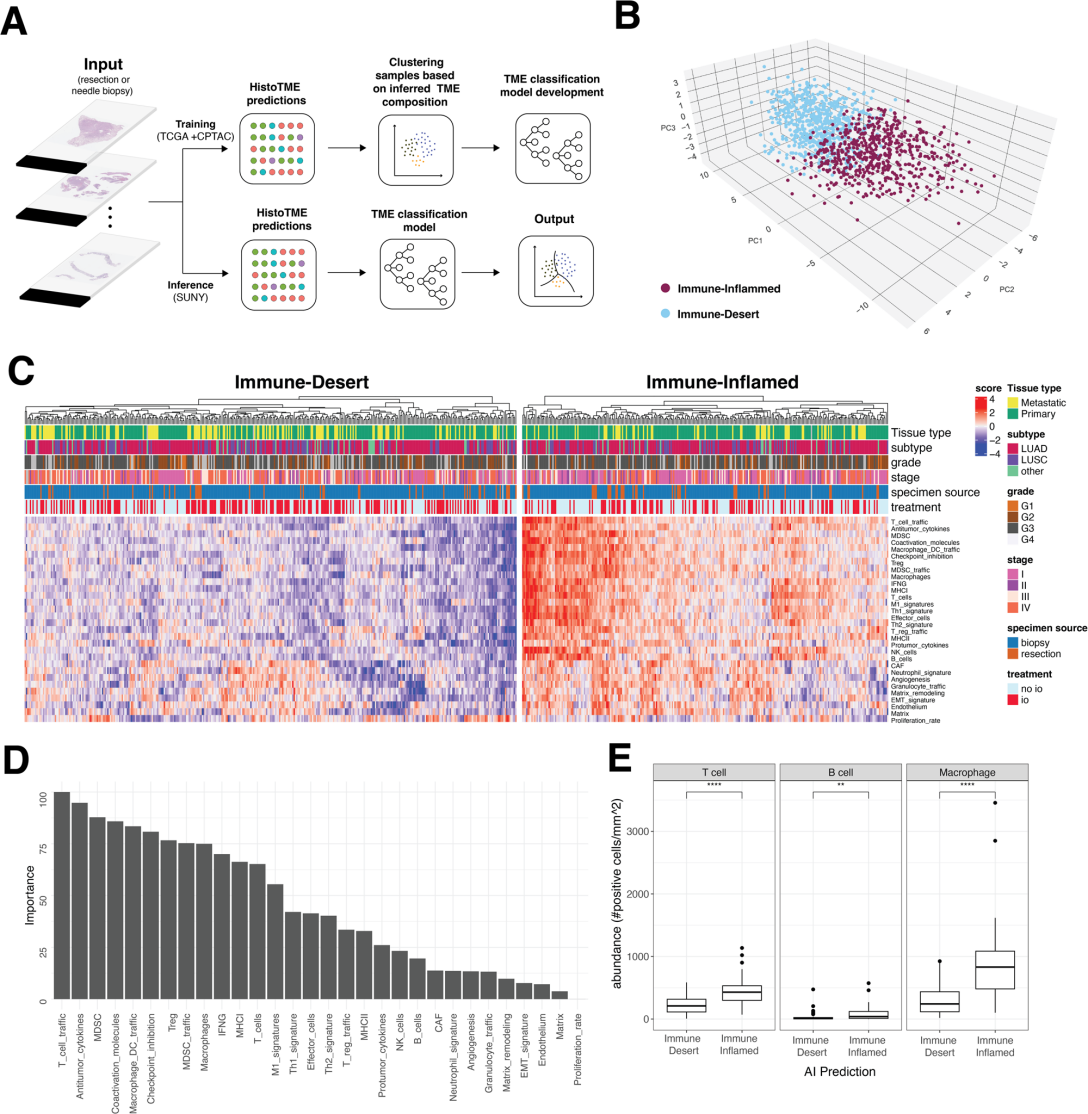
Overview of the computational pipeline to classify patients into distinct clusters based on their H&E-predicted TME composition. **A** H&E stained digitized tumor samples from TCGA + CPTAC are processed by HistoTME and subsequently clustered into two clusters based on partition around medoid (PAM) clustering and a Random Forest classification model that is trained on cluster membership data. **B** 3D PCA plot visualizing the two distinct clusters of TCGA + CPTAC NSCLC patients: Immune Inflamed and Immune Desert, based on their HistoTME-inferred TME profiles. **C** Heatmap depicting the H&E-predicted TME composition and clinical attributes of NSCLC patients from the SUNY cohort. Patients were classified into Immune Inflamed cluster or Immune Desert cluster using a two class classification model (Random Forest) trained on TCGA + CPTAC data. **D** Random forrest-derived feature importance rankings of TME signatures driving the distinction between the Immune Inflamed and Immune Desert cluster. **E** Immunohistochemistry-derived T cell, B cell and Macrophage abundances, defined as number of marker positive cells per mm^2^, in cases belonging to the immune-inflamed or immune desert cluster. Cell type abundances were quantified from whole slide immunohistochemistry images using QuPath v0.5.0 positive cell detection and quantification pipline. Statistical significance between groups was determined by non-parametric Wilox rank sum test (***p-value < 0.001, ****p-value < 0.0001).

We then trained a simple unsupervised model on the TCGA + CPTAC cohorts to cluster NSCLC patients into distinct subgroups based on their predicted TME signatures (Fig. 2A, see Methods). This model effectively identifies two main clusters resembling the Immune-Inflamed and Immune-Desert phenotypes^42–44^(Fig. 2B, Supplementary Fig. 4). Subsequently, we applied this two-cluster model to the entire institutional SUNY Upstate cohort, including patients with core needle biopsies (*N* = 652 patients) (Fig. 2C). We next compared the distribution of various clinical characteristics of patients across model-predicted immune inflamed and immune desert clusters including age, smoking history, tissue type (Primary vs metastatic), specimen source (biopsy vs surgical specimen), disease stage, grade, PDL1 score and histopathologic subtype. Overall, we do not find any significant differences in the distribution of majority of the clinical features across the two subtypes with the exception of tumor stage, tissue type (primary vs metastatic) and PD-L1 scores (Supplementary Fig. 5).

Upon examining the feature importance scores assigned to each TME signature by the trained two-class classification model (Fig. 2D), we found that the following signatures: T cell traffic, Antitumor Cytokines, MDSC(myeloid-derived suppressor cells), Co-activation molecules and Macrophage/Dendritic Cell Traffic were among the top 5 TME signatures driving the distinction between the Immune-Inflamed and Immune-Desert clusters. Interestingly, in line with these results, we noted substantial differences in the abundances of T cells, B cells and, macrophages, when observed through IHC, among patients predicted to be either in the Immune Inflamed or in the Immune Desert cluster (Fig. 2E).

**Fig. 3:**
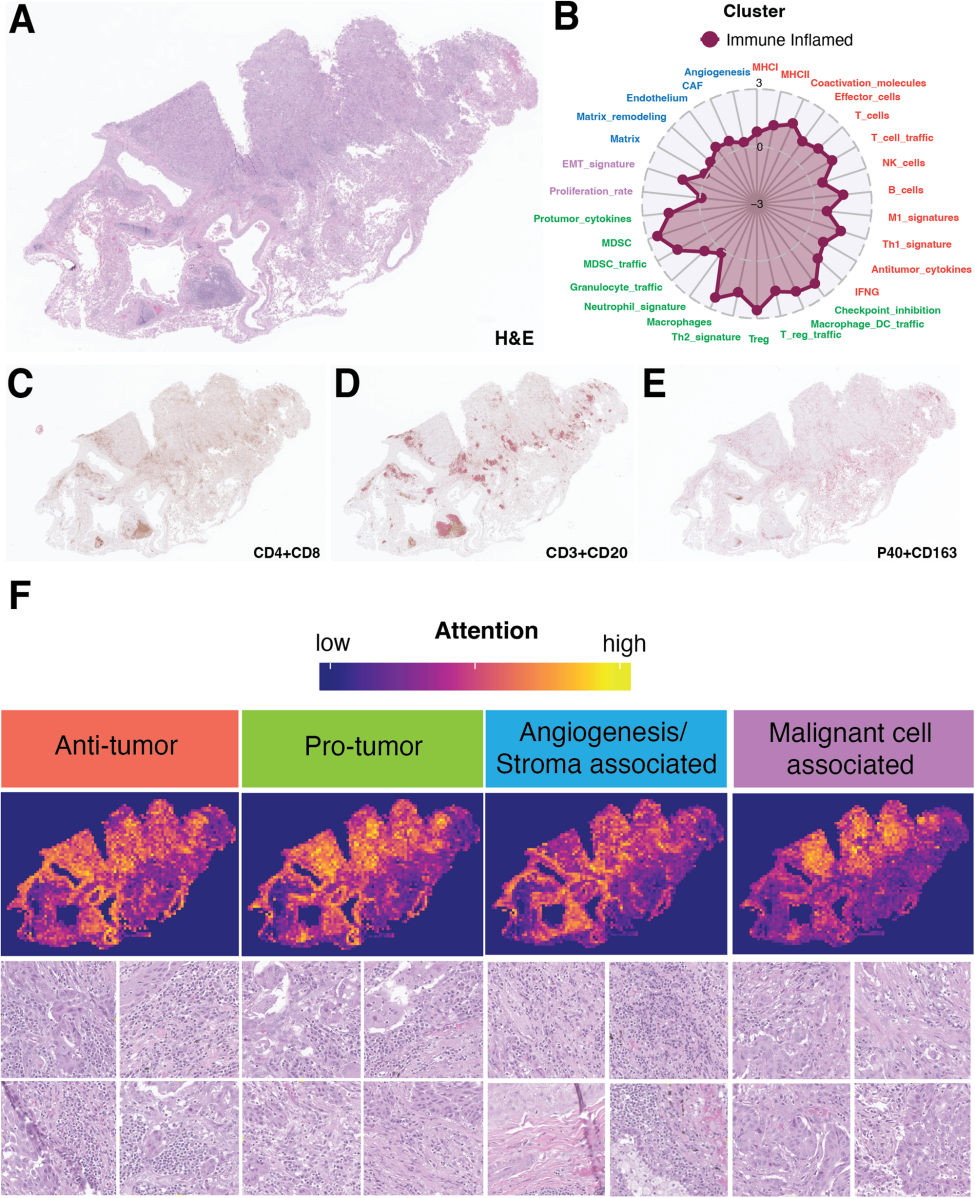
Overview of HistoTME predictions for a lung resection case predicted to be immune inflamed. **A**, **B** Low magnification view of a primary NSCLC tumor resection sample and its predicted TME signature profile. **C**–**E** Matched whole slide immunohistochemistry images of the same tumor sample dual stained for CD4 (brown)+CD8 (magenta), CD3 (brown)+CD20(magenta), and P40 (brown)+CD163 (magenta) markers respectively. **F** HistoTME generated attention maps for each attention head. Below each whole slide attention map are 4 high magnification image tiles (50 × 50 µm) randomly sampled from high attention areas. Supplementary Fig. 6 shows another related example along with higher magnification image tiles randomly sampled from high attention areas.

**Fig. 4:**
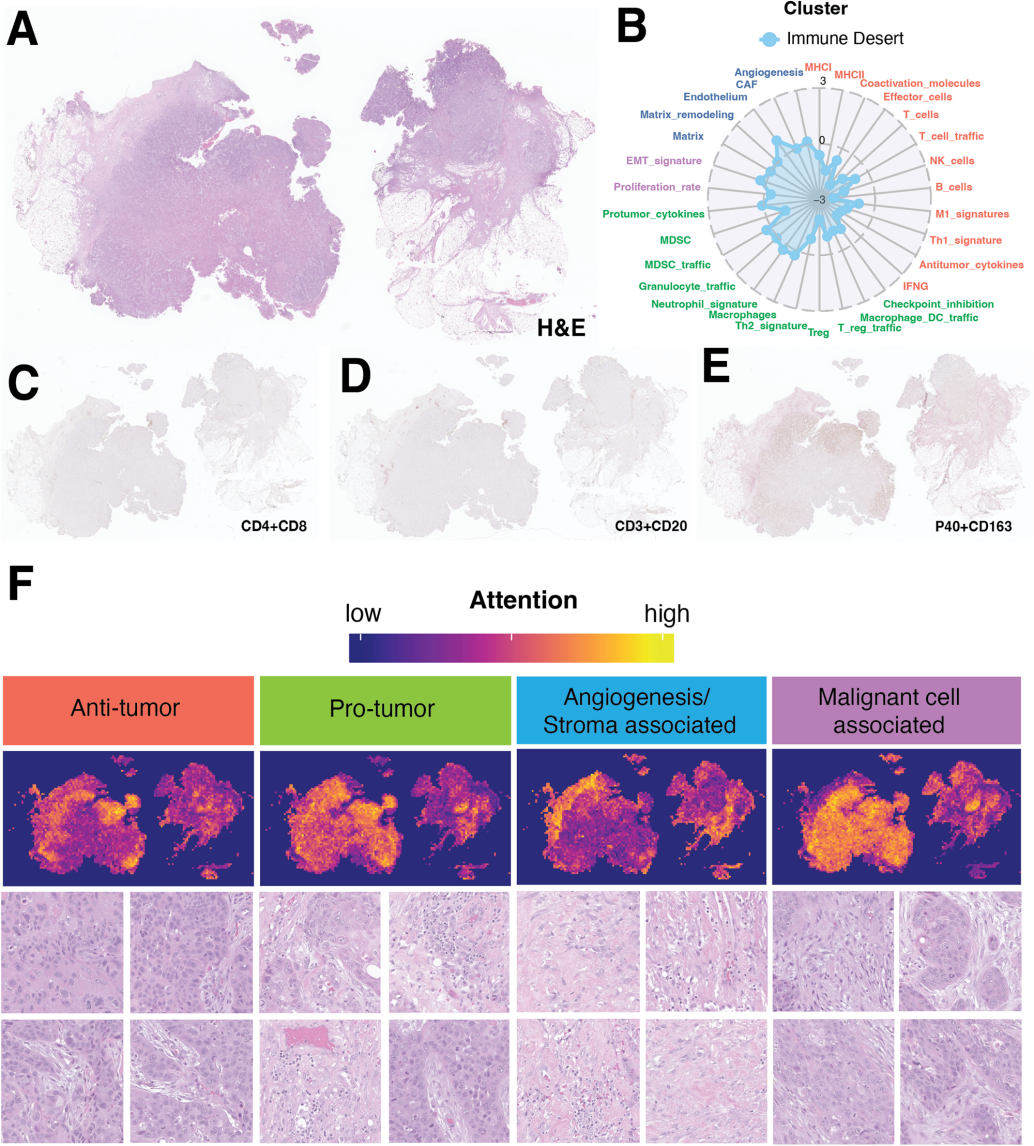
Overview of HistoTME predictions for a lung resection case predicted to be immune desert. **A**, **B** Low magnification view of a metastatic NSCLC tumor resection sample and its predicted TME signature profile. **C**–**E** Matched whole slide immunohistochemistry images of the same tumor sample dual stained for CD4 (brown)+CD8 (magenta)markers, CD3 (brown)+CD20(magenta) markers, and P40 (brown)+CD163 (magenta) markers respectively. **F** HistoTME generated attention maps for each attention head. Below each whole slide attention map are 4 representative high magnification image tiles (50 × 50 µm) sampled from high attention areas. Supplementary Fig. 7 shows another related example along with higher magnification image tiles randomly sampled from high attention areas.

To understand the histopathological features influencing HistoTME predictions, we generated attention maps for all 652 patients within the SUNY Upstate cohort. In general, HistoTME attends to different areas of the TME to estimate the expression of antitumor, pro-tumor, angiogenesis/stroma and malignant cell signatures, with the exception of antitumor and protumor immune signatures (Supplementary Fig. 6). We additionally performed a qualitative review of four randomly chosen resection cases (2 Immune-Inflamed, 2 Immune-Desert) with the help of two board-certified pathologists (Figs. 3 and 4, Supplementary Figs. 7 and 8). Overall, for immune-inflamed cases, which have relatively high predicted expression levels of antitumor and protumor immune signatures, HistoTME assigns great attention to regions abundant with lymphocytes and the formation of lymphocytic aggregates around tumor-stroma boundaries. For, immune desert cases, which have relatively low predicted expression levels of antitumor and protumor immune signatures, HistoTME assigns great attention to regions containing solid areas of more pleomorphic cells, and in addition, dense fibrotic areas within and around the tumor periphery. Collectively, these results suggest that HistoTME effectively captures the TME composition of tumors from H&E slides.

### Association between the tumor microenvironment status and survival outcomes of NSCLC patients treated with immune checkpoint inhibitors

We next conduct a retrospective analysis to assess how the TME status (Inflamed vs Desert), inferred from H&E slides, relates with survival outcomes of NSCLC patients from the SUNY Upstate cohort treated with immune checkpoint inhibitor therapy. All patients that underwent independent PD-L1 IHC testing were considered for treatment with immune checkpoint inhibitors with a total of 292 patients ultimately receiving either an anti-PD1 or PD-L1 inhibitor as monotherapy or in combination with chemotherapy. Overall, 77% of these 292 patients had PD-L1 ≥ 1% and 50% had metastatic disease (stage IV). A detailed summary of the clinical cohort is available in Supplementary Table 1.

**Fig. 5:**
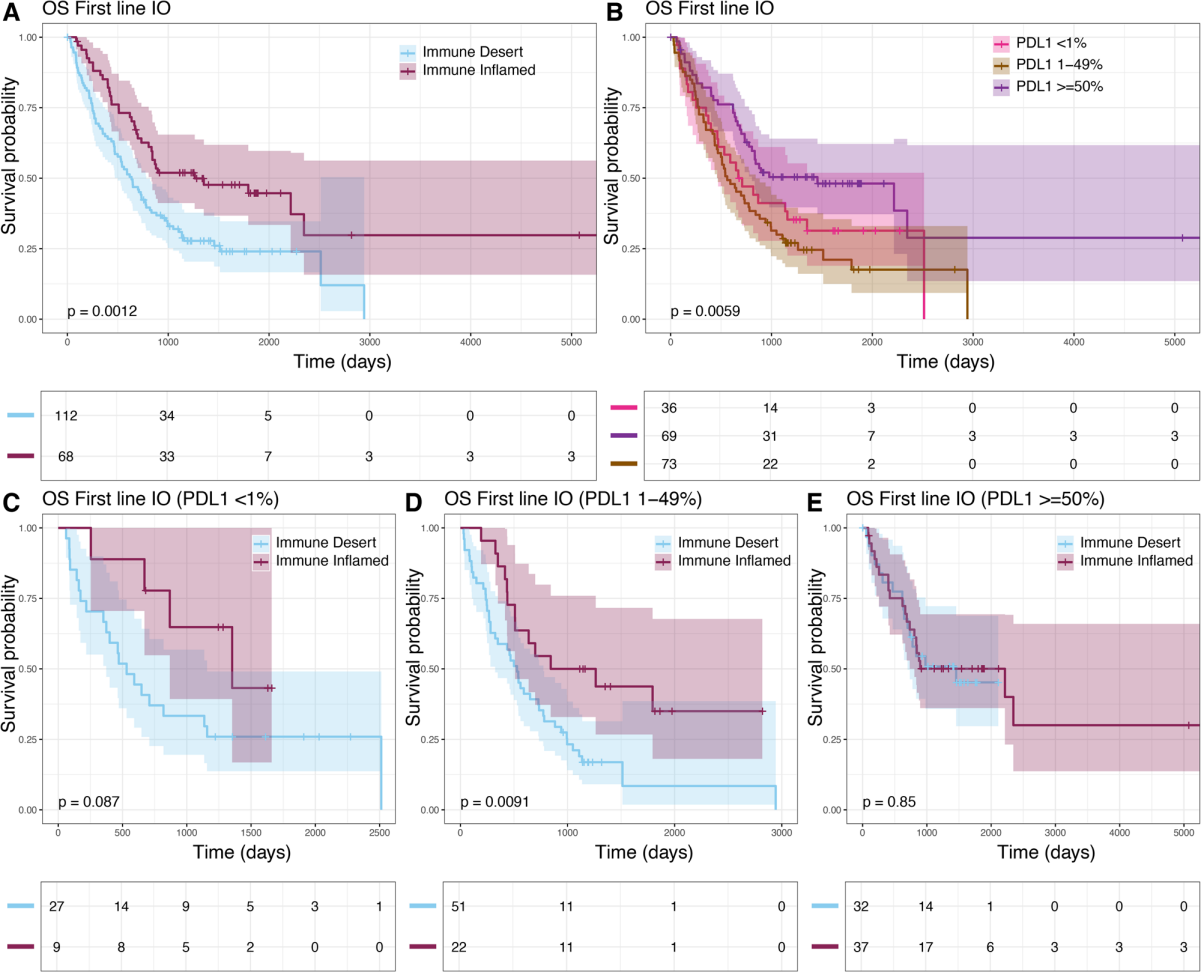
Association between HistoTME-based TME classification and overall survival outcomes of SUNY NSCLC patients treated with first-line anti-PD1/PD-L1 therapy (first-line IO patients). **A** Kaplan–Meier plot depicting overall survival−defined as time from date of diagnosis to date of death—of patients that received first-line anti-PD1/PD-L1 treatment. **B** Kaplan–Meier plot depicting overall survival of SUNY patients that received first-line anti-PD1/PD-L1 therapy stratified by PD-L1 IHC expression. **C**–**E** Kaplan–Meier plots depicting overall survival of first-line patients in PD-L1 negative (TPS < 1%), PD-L1 low (TPS = 1–49%) and PD-L1 high (≥50%), cases. Significance of survival differences between distinct subgroups of patients was determined by the log-rank test.

Interestingly, we found that the TME status is particularly predictive of overall survival of patients receiving ICIs as first line of therapy (Fig. 5A, log-rank test *p*-value = 0.0012, HR = 0.53 [95% CI: 0.36-0.78]), especially when administered in combination with chemotherapy (First line ICI+chemo log-rank test *p*-value: 0.00067, HR = 0.39 [95% CI: 0.22-0.68], First line ICI monotherapy log-rank test *p*-value: 0.22, HR = 0.68 [95% CI: 0.37-1.26]; Supplementary Fig. 9). Importantly, TME status remains predictive of overall survival of first line ICI-treated patients even after accounting for confounding effects of tumor stage, tissue type (primary vs metastatic) and PDL1 scores (HR = 0.57 [95% CI: 0.38-0.85], Cox proportional hazards *p*-value: 0.006; Supplementary Fig. 10) and restricting the analysis to patients with needle biopsy specimens (log-rank test *p*-value 0.0034, HR = 0.54 [95% CI: 0.36-0.82], Supplementary Fig. 11). The TME status, however, is less effective at predicting overall survival when considering all lines of ICI-treated patients (Supplementary Fig. 12A, log-rank test *p*-value: 0.02, HR = 0.7 [95% CI: 0.52-0.95]) and non-ICI treated patients (Supplementary Fig. 13, HR = 0.84 [95% CI: 0.6-1.19], log-rank test *p*-value: 0.029, Cox proportional hazards *p*-value: 0.34). When looking at PD-L1 expression, patients with PDL1 expression > = 50% and receiving ICI treatment as first line of therapy showed markedly improved survival compared to those with PD-L1 1-49% or <1% (Fig. 5B, log rank test *p*-value = 0.0059, HR = 0.62 [95% CI: 0.37- 1.04]). PDL1 expression was however less effective at predicting of overall survival when considering all lines of ICI-treated patients (Supplementary Fig. 12B, log rank test *p*-value = 0.13, HR = 0.9 [95% CI: 0.68–1.18]). When looking at progression-free survival (PFS), we observe that both the TME status and PD-L1 expression are primarily predictive of PFS at first line ICI therapy (TME status: log rank test *p*-value = 0.0037), HR = 0.59 [95% CI: 0.41-0.85]; PD-L1 expression: log rank test *p*-value = 0.003, HR = 0.55 [95% CI: 0.35-0.88]; Supplementary Fig. 14.

When performing additional subgroup analysis of patients receiving first line ICI treatment, we find that the H&E-inferred TME status is primarily predictive of overall survival outcomes of PD-L1 absent (<1%) patients (Fig. 5C; log rank test *p*-value = 0.08, HR = 0.4 [95% CI: 0.13- 1.18]), and PD-L1 low (1–49%) patients (Fig. 5D; log rank test *p*-value = 0.009, HR = 0.44 [95% CI: 0.23-0.82]) but not PD-L1 high (≥50%) patients (Fig. 5E; log rank test *p*-value = 0.85, HR = 0.94 [95% CI: 0.48-1.84]). Subgroup analyses by histologic subtype revealed that HistoTME-derived TME status was particularly predictive of overall survival in adenocarcinoma patients, which were a majority of the patients treated with first line ICI therapy, while less so in patients with squamous cell carcinoma (Supplementary Fig. 15A, B). Interestingly, first-line ICI patients with rare neuroendocrine or pleomorphic histologic subtypes (i.e., defined as “other”) and an immune-inflamed TME had worse overall survival compared to those with an immune-desert TME in first line ICI treatment setting (Supplementary Fig. 15C).

When utilizing the TME status to predict treatment responses (i.e., predict immune inflamed as responder and immune desert as non-responder), we achieve a sensitivity of 0.46 [95% CI: 0.35-0.58], specificity of 0.72 [95% CI: 0.60-0.81] and positive predictive value of 0.64 [95% CI: 0.51-0.76] for first line ICI-treated patients and a sensitivity of 0.44 [95% CI: 0.35-0.54], specificity of 0.73 [95% CI: 0.63-0.81] and positive predictive value of 0.63 [95% CI: 0.52-0.74], when considering all ICI-treated patients. When utilizing PD-L1 expression to predict treatment response (i.e., predict PD-L1 > = 50% as responder and PD-L1 1-49% or <1% as non-responder), we achieve a sensitivity of 0.49 [95% CI: 0.38-0.60], specificity of 0.74 [95% CI: 0.63-0.84] and positive predictive value of 0.67 [95% CI: 0.54-0.79] for first line ICI-treated patients and sensitivity of 0.44 [95% CI: 0.35-0.54], specificity of 0.72 [95% CI: 0.62-0.80] and positive predictive value of 0.63 [95% CI: 0.51-0.73] when considering all ICI-treated patients. When combining TME status and PD-L1 expression into a single predictor, which defines any patient with either an inflamed TME or PD-L1 > = 50% as responders, we achieve a sensitivity of 0.69 [95% CI: 0.58-0.79], specificity of 0.59[0.47-0.70] and positive predictive value of 0.64 [95% CI: 0.54-0.74] for first line ICI-treated patients and a sensitivity of 0.65 [95% CI: 0.56-0.74], specificity of 0.56 [95% CI: 0.47-0.66] and positive predictive value of 0.62 [95% CI: 0.52-0.70] when considering all ICI-treated patients. Collectively, these results highlight the complementary value of HistoTME for prognostication of NSCLC patients, especially for those with PD-L1 < 50% and being considered for first line treatment with ICI+chemotherapy.

Besides PDL1, several groups have also explored the prognostic value of tumor infiltrating lymphocytes (TIL) detected from histopathology images, using automated deep learning tools^27,45,46^. Hence, we evaluated the added prognostic value of HistoTME-derived TME signatures relative to percentage TILs detected using a state-of-the-art open source TIL detection tool, CellVIT^47^, with well documented source code for cell segmentation and classification. Immune inflamed patients had significantly higher %TIL counts relative to non-inflamed patients (Supplementary Fig. 16A) highlighting the ability of HistoTME to capture variations in immune cell abundances, despite being trained without any pathologist annotations. We next aimed to assess the prognostic value added by HistoTME-derived immune inflamed and immune desert clusters upon %TILs derived from CellVIT. Hence, we first stratified patients treated with first line ICI into high-TIL and low-TIL subgroups based on a previously established cut-off of >10% TILs in NSCLC^48^ and then compared overall outcomes of each %TIL subgroup. Our findings indicate that HistoTME-derived immune inflamed and immune desert predictions achieve superior stratification of overall survival outcomes of patients receiving first line ICI therapy compared to %TIL subgroups (Supplementary Fig. 16B), and can predict ICI responses in both low and high %TIL subgroups (Supplementary Fig. 16C, D). Moreover, joint Cox proportional hazards modeling of both HistoTME and CellVIT revealed that HistoTME-predicted immune inflamed and immune desert clusters achieve statistically significant hazard ratios, exceeding the magnitude of hazard ratios achieved by %TILs (Supplementary Fig. 16E). These results collectively highlight the added prognostic value of a comprehensive assessment of the TME composition, beyond TILs alone, for accurate prediction of patient responses to ICI therapy.

### Interactions between TME signatures improve prediction of immunotherapy response

Interactions among the various components of the TME play a key role in influencing immunotherapy responses^49^. A previous study by Liu et al. highlighted an example of this complexity, revealing in NSCLC patients treated with ICI that only high PD-L1 expression in macrophages was correlated with better overall survival, while high PD-L1 expression in tumor or stromal cells was not^17^. Hence, we developed a supervised ML model that incorporates interactions between H&E-inferred TME signatures to predict immunotherapy responders (See Methods).

**Fig. 6:**
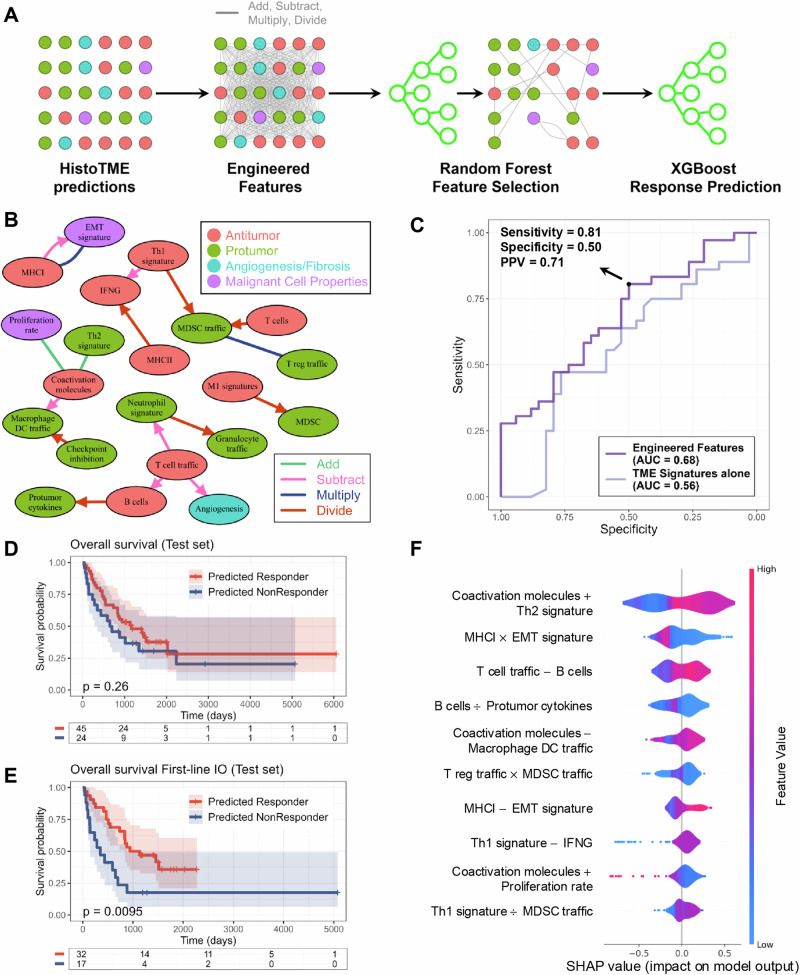
Overview of supervised machine learning model development and validation for predicting immune checkpoint inhibitor responses based on HistoTME predictions. **A** Model development for response prediction: (1) HistoTME predictions are engineered into new features by taking pairwise sums, differences, products, and quotients. (2) random forest feature selection. (3) XGBoost trained for response prediction. **B** Feature network pairwise interactions of 18 selected features. Arrow endpoints denote the signature subtracted or divided from the signature at the start point. **C** Test set receiver operating characteristic (ROC) curve of the model trained on engineered features or TME signatures alone. Optimal cut point shown based on the Youden index. Kaplan Meier plot depicting overall survival of the test set stratified by AI response prediction for **D** all patients that received anti-PD1/PD-L1 treatment and **E** first-line immunotherapy (IO)-treated patients. **F** Shapley additive explanation (SHAP) summary plot ordered by SHAP importance.

Specifically, we engineered 1740 interaction features by taking the sum, difference, product, and quotient of each pair of signatures to characterize interactions between TME signatures, then used a random forest model to select the most important interaction features from the training set, and trained XGBoost, a gradient boosted decision tree, using selected interaction features for ICI response prediction (Fig. 6A; Methods). After applying 5-fold cross-validation to the training set to optimize the number of features selected, 18 TME signature interactions were chosen. These interactions maximize the cross-validation receiver operating characteristic curve (AUROC), achieving a CV AUROC of 0.68 (Supplementary Fig. 17). Of the 18 pairwise interactions, coactivation molecules, T cell traffic, and MDSC traffic were incorporated most frequently (Fig. 6B). We then retrained XGBoost on the full training set using these 18 selected interaction features. The trained model utilizing interaction features predicted response on the test set with an AUROC of 0.68 [95% CI: 0.55-0.80], whereas using TME signatures alone, without interactions, achieved an AUROC of 0.55 [95% CI: 0.41-0.69], although the difference was not significant (*p* = 0.17, paired DeLong’s test) (Fig. 6C). Furthermore, when only considering patients from the test set that received first line ICI-treatment, we found accuracies for predicting treatment response improved to an AUROC of 0.75 [95% CI: 0.61-0.88], while achieving a lower AUROC of 0.51 [95% CI: 0.23-0.78] for non-first-line ICI-treated patients.

At a probability threshold that maximized the Youden index, the model predicted responders with a sensitivity of 0.80 [95% CI: 0.63-0.92], specificity of 0.50 [95% CI: 0.32-0.68], and positive predictive value of 0.71 [95% CI: 0.49-0.87]. At this threshold, the predicted responders did not have significantly higher overall survival compared to non-responders in the entire test set (OS: HR = 0.35; 95% CI: -0.26 to 0.96; *p* = 0.26). However, when considering only first-line ICI-treated patients in the test set, the predicted responders had significantly longer survival time compared to non-responders (OS: HR = 0.90; 95% CI: 0.20 to 1.60; *p* = 0.0095) (Fig. 6D, E). Furthermore, we observed significantly higher progression-free survival (PFS) outcomes for patients that were treated with first line ICI and predicted to be responders (PFS: HR = 0.94; 95% CI: 0.29 to 1.59; *p* = 0.0035) but not for second-line or subsequent-line ICI-treated patients (Supplementary Fig. 18). Using a Shapley additive explanation (SHAP)^50^ summary plot ordered based on feature importance, we determined TME signature interactions that had the most influence on the response prediction, with the most important interaction consisting of the sum between coactivation molecules and Th2 signatures (CM+Th2) (Fig. 6F). High levels of CM+Th2 corresponded to higher SHAP values, indicating higher predicted probabilities of response.

## Discussion

Immune checkpoint inhibitors have emerged as a promising treatment option for lung cancer. Yet only a minority of patients respond to these treatments, sometimes at the cost of severe toxicities and financial implications for those who may not respond to this therapy. Hence it is imperative to identify effective biomarkers capable of differentiating responders from non-responders prior to therapy initiation. The tumor microenvironment plays a fundamental role in shaping the responses of patients to immunotherapy. However, its dynamic nature, with multiple interacting components, makes it challenging to identify robust predictive biomarkers^32,33^. The integration of deep learning methods with digital pathology presents a promising and potentially cost-effective approach to interrogate the TME and alleviate some of these issues^51^.

In this work, we introduce HistoTME, a novel weekly supervised deep learning method to characterize the TME of patients from H&E slides, leveraging enhanced feature extraction capabilities of recent digital pathology foundation models. In contrast to recent deep learning approaches, which aim to predict spatially resolved gene expression profiles from histopathology images^52–55^, our approach aims to infer the TME composition through estimating the expression of distinct functional TME signatures. A key advantage of this approach is that by learning to predict the expression of gene signatures, we not only avoid overfitting to the expression of individual genes but also increase interpretability by directly relating specific histopathological features to previously established biological concepts^38^. Due to the limited benchmarking of foundation models for continuous biomarker prediction tasks, we experimented with three popular foundation models as feature extractors. We found out that the UNI foundation model^32^, when paired with multi-task AB-MIL, achieved the best predictive performance for the various TME signature prediction tasks. This improved performance likely stems from the considerably large histopathology datasets used to pre-train the UNI foundation model, as well as the added regularization induced by multitask learning.

With the help of HistoTME, we next classify patients into two distinct TME subtypes: Immune-Inflamed and Immune-Desert, characterized by distinct expression of anti-tumor and protumor immune signatures. These observations were further corroborated by significant differences in cellular abundances of T cells, B cells and macrophages across patients, based on matched IHC data. Moreover, the two distinct TME subtypes were significantly predictive of OS and PFS in the patients treated with first-line immune checkpoint inhibitors, going beyond TIL estimates and PD-L1 scores. While numerous deep learning studies have emerged for predicting ICI responses in NSCLC from H&E images, they are primarily focused on refining PD-L1 quantification^56–58^. In contrast to previous studies, our approach aims to offer a more comprehensive overview of the tumor microenvironment by predicting the TME cell type and molecular composition. The rationale and motivation behind our approach come from recent clinical trials showing that PD-L1 expression alone is insufficient to predict ICI treatment outcomes (Checkmate 057^59,60^, OAK^61^). Our results indicate that HistoTME-generated TME composition predictions can further identify patients associated with ICI response, particularly in the low PD-L1 expression group where additional biomarkers for ICI response are urgently needed. In the future, we anticipate that combining our approach with emerging deep learning-based PD-L1 biomarker assays will further enhance ICI response prediction.

In addition to clustering, we developed a novel supervised model to predict ICI response, which analyzes interactions between distinct TME signatures. Our supervised model achieves an AUROC of 0.68 in the unseen test patient cohort, improving to an impressive AUROC of 0.75 when predicting responses to first-line immunotherapy, highlighting the significance of interactions among various TME components in shaping treatment responses. On performing SHAP analysis, we found that the total expression of coactivation molecules and the Th2 signature were the most predictive of ICI response. This observation likely stems from the importance of coactivation molecules, such as CD28, which is essential for T cell activation after interacting with antigen-presenting cells, in cancer immunotherapy response^62^. Although Th2 signatures have been associated with an immune suppressive TME, their role in responses to ICI treatment remains unclear^63^. The emphasis laid by the AI model on this interaction could be explained by coactivation molecules promoting production of Th2 cytokines^64^, indicating that high levels of coactivation molecules and Th2 may offer a favorable environment crucial for therapeutic response. It is important to note, however, that interpreting the feature importance of ML models requires caution due to potential variability in feature importance depending on the training set, particularly with limited-size datasets.

Compared to previous studies of predicting response to ICI from H&E slides, we demonstrated a unique ability to identify responders using both unsupervised and supervised ML techniques. Both these approaches can be valuable for deriving ICI response biomarkers depending on the availability of clinically annotated datasets. Hu et al. developed a supervised deep learning model that used extracted hidden features from histopathology images to predict anti-PD-1 response in melanoma and lung cancer. However, a main caveat of their approach was the limited interpretability of their hidden features^65^. In addition, they reported modest accuracies (AUROC: 0.645 [95% CI: 0.495-0.784]) in predicting ICI response for lung cancer patients, which they attribute to the dataset consisting of core-needle biopsy samples rather than surgery samples. In another recent study, Wang et al. achieved an impressive performance by using hand-crafted features derived from spatial interactions of tumor cells and TILs^27^; however, their approach required a minimum of five large image patches due to the reliance on spatial interactions, which may not generalize well on needle biopsies. Most importantly, to our knowledge, this is the first study showing that predicting molecular features of TME is feasible from scanned H&E images. This makes HistoTME extremely versatile and useful for analysis of both surgical resection and needle biopsy data. In fact, HistoTME signatures are able to effectively predict responses of patients while also maintaining interpretability, despite limited availability of tumor tissue from core needle biopsies, which make up ~85% of the SUNY cohort.

This work has some limitations that should be further considered. First, while HistoTME offers valuable insights into TME composition from H&E slides, its inability to predict the spatial distribution of specific cell types or molecules is a limitation stemming from its weakly supervised training on bulk transcriptomics data. To address this, future advancements in HistoTME could consider harnessing emerging large-scale spatial transcriptomics datasets^66^ for enhanced training and validation. Second, although a large clinical cohort of ICI-treated patients was studied, we lacked additional external validation datasets to further validate the prediction of responses to ICI. Second, this work was conducted in a retrospective manner. We plan to further validate HistoTME in additional external cohorts of patients treated with ICI, both retrospectively and prospectively. Third, the development of HistoTME was limited to patients with NSCLC, implying that further testing and development will be required to extend the approach to different cancer types. Fourth, only three TME signature predictions were validated using IHC due to the lack of other strictly comparable IHC markers for other signatures. Bagaev et al.^38^, who previously analyzed these TME signatures directly from transcriptomics data identified 4 distinct TME subtypes, which besides capturing differences in the activity of immune signatures also capture notable differences in activity of stromal and angiogenesis-associated signatures. In contrast, our approach results in the identification of two subtypes (Immune-Inflamed and Immune-Desert). This difference could potentially be explained by lower predictive accuracies of HistoTME for stroma and angiogenesis-associated signatures compared to immune signatures. We plan to utilize more complex molecular profiling tools, such as spatial transcriptomics, to further train and validate the accuracy of HistoTME predictions for other signatures. Lastly, while we showed promising results for predicting responses to first-line ICI treatment, the predictive accuracy of HistoTME was limited for the patients who received immune checkpoint blockade as second and subsequent-line treatment. This finding can be attributed to the fact that a majority of patients in this cohort received ICI as first-line treatment. In addition, since the TME is dynamically changing throughout the treatment, reflecting either response to therapy or tumor progression^67^, the H&E WSIs may not accurately represent the TME of patients following first-line treatment. Hence, it is important to consider the time interval between the H&E biopsy and ICI treatment to assess the utility of HistoTME for the prediction of treatment response.

In conclusion, HistoTME is an effective approach to characterize the TME of NSCLC patients and identify patients who will benefit from ICI therapy. Being based on H&E slides alone, HistoTME allows for a broad characterization of the TME without the need for expensive molecular tests or additional tissue stains. Given the routine use and low cost of H&E slides in diagnostic pathology along with the increasing adoption of digital and computation pathology in clinical practices, HistoTME promises to improve clinical management of cancer patients undergoing immunotherapy. Future research should focus on validating HistoTME in diverse patient populations and exploring its applicability to other cancer types, potentially extending its benefits beyond lung cancer. Finally, HistoTME can help advance our understanding of the role of TME in the context of other cancer treatments, opening avenues for the discovery of novel biomarkers and accelerating the adoption of personalized immuno-oncology.

## Methods

### Description of the SUNY NSCLC cohort

This retrospective institutional cohort of 692 NSCLC patients was assembled at SUNY Upstate university, of which 652 were analyzed in this study based on the following criteria: primary diagnosis of non-small cell lung cancer who were followed at SUNY Upstate for at least a minimum of 2 years, and had PD-L1 testing record along with IHC slide availability including corresponding H&E slides (*n* = 652 patients, 1329 H&E slides). Neoadjuvant or perioperative cases were not included and analyzed in our study. All H&E slides from the SUNY Upstate cohort were obtained prior to receiving any treatment. The query and chart review were done using electronic medical records (EMR, EPIC) and pathology information system (Co-Path) for abstracting clinico-pathological, treatment and follow-up information. All clinical and pathology imaging data from the SUNY Upstate cohort was approved for use in this study by the local Institutional Review Board (reference numbers: 1857564, 2153970) and considered exempt from further IRB oversight. The requirement for patient consent was waived as all data was collected retrospectively and anonymized. The details of the cohort description are provided in Supplementary Table 1. Briefly, 292 (44.8%) patients were treated with immunotherapy, and of these patients, 230 had treatment response information (partial, complete, stable) as documented radiographically or clinically by the treating physician in the patient’s charts (progress notes, EPIC). More specifically, the responders were defined as patients that exhibited a partial, complete, or stable disease without experiencing any recurrence or death for at least 6 months since the start of ICI treatment. Non-responders were defined as patients who exhibited progressive disease or death within 6 months since the start of ICI treatment. Overall survival (OS) of patients was defined as the time from the date of diagnosis until death from any cause. Patients who were alive at the last follow-up were censored for overall survival analysis. Progression-free survival (PFS) of patients receiving checkpoint inhibitor treatment was defined as the time interval from immune checkpoint inhibitor start until progression or death. Patients who were alive without disease progression at their last follow-up were censored for PFS.

### Specimen availability, immunohistochemical analysis and slide scanning

Briefly, from 652 patients, 445 had tumor specimens available from primary disease sites (biopsy = 398, resection = 47) and 207 from metastatic sites (biopsy=169, resection = 35). Serial immunostaining was done on four-micrometer-thick freshly cut serial sections using archived FFPE blocks of surgically resected specimens from 82 cases diagnosed with NSCLC (47 primary sites, 35 metastatic sites). The following biomarker panel was used for immune (CD3, CD20, CD4, CD8, CD163, FOXP3), cancer-specific (TTF-1, P40) and epithelial markers (PAN-CK). The staining was performed in a CLIA-certified clinical pathology lab using an automated immunostainer BenchMark Ultra (Roche Diagnostics, Germany) at SUNY Upstate Medical University. For pretreatment, antibody detection and counterstaining, the following reagents were used: ULTRA CC1 (Cat #950-124), UltraView DAB (Cat. 760–500), UltraView Red (Cat # 760-501) and Hematoxylin (Cat. 760–2021) according to the manufacturer’s instructions (Ventana Medical Systems; Roche Diagnostics, Germany). The details of the primary and secondary antibodies, antigen retrieval conditions, as well as detection methods are listed in Supplementary Table 2. PD-L1-stained slides (clone 22C3 PharmDx^TM^, 28-8 pharmDx^TM^, Dako Autostainer Link 48 platform), along with negative controls and corresponding H&E of 406 patients, were requested from LabCorp and the rest (*n* = 246 patients) were obtained from the local pathology archives at SUNY Upstate Medical University. Glass slides were digitized using an Aperio AT2 Dx scanner (Leica Biosystems, CA, USA) at 40x magnification at the Pathology Research Core at SUNY Upstate. PD-L1 manual scoring was performed by expert pathologists using an FDA-approved assay and scoring guidelines at LabCorp. Tumor proportion score (TPS) was calculated as the % of viable positive tumor cells/all tumor cells, where positivity was defined as partial and/or complete membrane staining at any intensity ( > 1%) in tumor cells. PD-L1 quantification was categorized into clinically relevant groups as approved by the FDA: <1% (absent), 1-49% (low), and ≥50% (high).

### Description of pre-processing steps for whole slide H&E images

All WSI in the experiments were first preprocessed to mask out the tumor tissue from background using RGB to HSV color transformation, median blurring and Otsu thresholding^68^. Following tissue segmentation, each WSI was split into image tiles of physical size 256 µm × 256 µm (i.e 512 × 512 pixels at 20x magnification). Our choice of tessellation of whole slide images at this resolution was motivated by recent successful applications of foundation models on H&E slides, as demonstrated by studies predicting colorectal cancer MSI^69^ and pan-cancer homologous recombination deficiency^70^. Each whole slide image tile with an overlap >25% with the tumor tissue was stain normalized using the Macenco algorithm^71^ and scaled to have 0 mean pixel intensity and standard deviation of 1 prior to being fed as input to an open-source foundation model—CTransPath^33^, RetCCL^37^ or UNI^32^—which learns to extract informative histopathologic features from each tile. CTransPath model consists of a convolutional neural network (CNN) and a multi-scale Swin Transformer architecture as its backbone, RetCCL uses a CNN-based architecture, and UNI implements a vision transformer (ViT). All foundation models were pre-trained self-supervised learning; CTransPath and RetCCL were pretrained on ~30,000 WSIs, while UNI was pretrained on ~100,000 WSIs. Feature extraction from each pretrained foundation model results in a feature matrix of shape *(n* *×* *768)* for CTransPath, *(n* *×* *2048)* for RetCCL, and (*n* *×* *1024)* for UNI per patient, where n represents the number of total number image tiles derived from WSI of each patient.

### Experimental setup and implementation details of HistoTME

HistoTME was trained using matched patient-level bulk RNA sequencing and whole slide imaging data of 865 patients (955 WSIs) from the TCGA cohort and validated on patient-level bulk RNA sequencing and whole slide imaging data of 333 patients (1501 WSIs) from the CPTAC cohort. Pre-processed bulk RNA sequencing data (gene level TPM counts) from each patient were downloaded from NCI Genomic Data Commons and further analyzed using the bioinformatics pipeline previously published by Bagaev et al. to calculate the average normalized expression of distinct functional gene sets^38^, referred to as *TME signatures*, which comprehensively capture TME composition and its various functional characteristics.

For each patient, histopathologic features were extracted from WSI tiles using one of the three foundation models described above. WSI tile-level features were then concatenated together into a single “bag-of-features” representation to facilitate weakly supervised regression, using the attention-based multiple instance learning (AB-MIL) method proposed by Ilse et al.^40^. The AB-MIL model consists of a learnable attention module, which assigns a weight, commonly referred to as *attention*, to each tile, and a feature aggregation module, which calculates the weighted sum of features across all tiles. This results in a single patient-level representation, summarizing key histopathological characteristics of the TME. The output of the feature aggregation module is then fed to a multilayer perceptron (MLP) module, which learns to predict the expression of a specific TME signature (single task) or multiple functionally related TME signatures (multi-task) as established previously by Ayers et al.^39^ and Bagaev et al.^38^. The AB-MIL model was trained with the AdamW optimizer^72^ and Huber loss function with delta set to one, which mitigates overfitting of model predictions to outliers by balancing the mean squared error and mean absolute error together, defined by Eq. (1):

1 $$L\left(y,\,f\left(x\right)\right)=\left\{\begin{array}{c}{\frac{1}{2}(y-f(x))}^{2}\\ \delta \left|y-f(x)\right|-\frac{1}{2}{\delta }^{2}\end{array}\right.\begin{array}{c}{for}\left|y-f\left(x\right)\right|\le \delta\\ {otherwise}\end{array}$$

The learning rate was set to 1 × 10^−4^ with a weight decay of 1 × 10^−4^. Batch size was set to 1 with gradient accumulation for 8 batches. Overall, for each model benchmarked in this study, training was done for 40 epochs with early stopping criteria of 10 consecutive epochs with no improvement in validation loss.

After completion of training, the Pearson correlation metric was utilized to evaluate accuracy of predictions of each model on the independent validation set (CPTAC-NSCLC). 95% confidence intervals (CI) of the Pearson correlation metric were estimated through 1000 bootstrapping iterations using the SciPy package^73^. The model achieving the highest Pearson correlation coefficients, on average, was eventually selected for external testing and determining ICI efficacy. All AI models were developed using open source PyTorch version 2.1.0^74^.

The external test cohort consisted of serial H&E and multiplex IHC sections of surgically resected tumors from 82 NSCLC patients (47 primary tumors, 35 metastases) enrolled at SUNY Upstate Medical University (See cohort description above). The best model, as determined from the benchmarking experiments on the CPTAC validation set, was applied to this test cohort to estimate the expression of T cell, B cell and Macrophage signatures. These expression predictions were then compared against the actual cellular abundances of respective cell types from corresponding IHC slides, using both Pearson and Spearman correlation metrics given the different scales of quantification. Cellular abundances of T cells (CD4, CD8), B cells (CD20) and Macrophages (CD163) were estimated from corresponding IHC stains using an open-source cell counting software QuPath (v0.5.0)^75^. Specifically, for each whole-slide IHC image, the tumor tissue was manually segmented and separated from the background. Following manual segmentation, a standard pipeline of stain deconvolution and positive cell segmentation was implemented to quantify the cell type abundance, defined as the total number of marker-positive cells per mm^2^ (https://qupath.readthedocs.io/en/stable/docs/tutorials/cell_detection.html). Three patients from this test cohort contained surgical resections from lymph-node metastases with an extremely high number of immune cells and were excluded from correlation calculations, to avoid reporting inflated accuracies.

### Predicting TME status using HistoTME

K means clustering algorithm was utilized to cluster patients from the TCGA and CPTAC-NSCLC cohorts into distinct TME subtypes based on HistoTME-predicted microenvironment signatures. Optimal number of clusters for K means clustering was determined using the average silhouette score metric^76^ (Supplementary Fig. 4), which revealed 2 distinct TME subtypes. To ensure the robustness of our findings, we conducted a bootstrap analysis, repeatedly applying the silhouette scoring method to clustering results generated from different randomly sampled subsets of 80% size of the original dataset. This analysis generated an empirical distribution of silhouette scores for each value of K, allowing us to assess the robustness of cluster assignments. The highest silhouette scores consistently occurred for *K* = 2, providing strong evidence for the existence of two distinct clusters in our data. Finally, a random forest classification model was trained on the clustered data to enable classification of individual patients from the SUNY cohort into distinct TME subtypes. To mitigate issues arising from data distribution shifts across cohorts, all predicted signature expression values were further scaled by their respective dataset-specific means and standard deviations prior to the development of the TME subtype classification model.

### Prediction of ICI response utilizing HistoTME-derived microenvironment signatures

Training and test sets were derived from 230 NSCLC patients with matched whole slide imaging data and treatment response labels as described above for supervised machine learning analysis using a stratified random splitting strategy (70% train, 30% test) implemented in scikit-learn package^77^. A total of 161 patients (84 responders, 77 non responders) were assigned to be part of the *IO training set* and 69 patients (35 responders, 34 non responders) were assigned to be part of the *IO test set*. A supervised machine learning model was trained to predict immunotherapy response using data from the IO training set. The model takes as input the 30 HistoTME predicted microenvironment signatures. In addition, 1740 interaction features were engineered by taking the sum, difference, product, and quotient of each pair of TME signatures. Since HistoTME signatures include negative and positive values, we take the exponent of each signature prior to computing the product and quotient to maintain monotonic relationships and preserve interpretability. A Random forest feature selection approach was used to select the top k most important features predictive of ICI response from the training set. A gradient-boosted decision tree model, XGBoost^78^, was trained using these selected features to predict ICI response. To identify the best set features for ICI response prediction, we utilized a 5-fold cross-validation strategy on the training data. Here, 80% of the training set was randomly allocated for model training, while the remaining 20% of the training set was reserved for validation purposes. A separate cross-validation iteration was conducted for each set of k features, and the number of boosting rounds was selected to optimize two metrics (minimize logistic loss and maximize AUROC) during cross-validation. Early stopping was set to 100 to select boosting rounds, where additional boosting rounds are not created if the metric does not improve after 100 rounds. The set of features that maximized the cross-validation AUROC was chosen as the final set of features. These features were then utilized along with XGBoost to train the final model on the entire training set. XGBoost learning rate was selected at 0.1, gamma was set to 0.1 to reduce overfitting, and the number of boosting rounds was set to 35 based on cross-validation. SHAP (Shapley Additive exPlanations) was used to interpret the output of the XGBoost model and estimate contribution of each feature^50^.

### Additional analyses and statistical tests

The Wilcoxon ranked sum test was used to determine the significance of differences in cellular abundances, as inferred from IHC, between the two predicted TME subtypes. The log-rank test was used to evaluate the prognostic significance of HistoTME in predicting PFS and OS. Response prediction was evaluated by AUROC. 95% Confidence intervals for AUROC were computed by the DeLong approach^79^. The optimal cutpoint of the ROC curve was chosen at the threshold that maximized the Youden index (J)^80^. 95% confidence intervals of precision, recall, and positive predicted value of the response predictions were calculated using exact binomial confidence limits. Kaplan-Meier survival curves were used to visualize the differences in the OS and PFS of AI-predicted responders and non-responders. Hazard ratios between survival groups with 95% CIs were calculated using a univariate Cox proportional hazards regression model. All statistical tests were two-sided, with a *p*-value less than 0.05 considered statistically significant. All statistical analyses were performed in R version 4.3.1 unless otherwise specified.

## Supplementary information

Supplementary Information

## Data Availability

The TCGA Whole slide Imaging and bulk transcriptomics data is publicly available and can be downloaded from the GDC portal (https://portal.gdc.cancer.gov). The CPTAC lung cancer whole slide imaging data is publicly available at The Cancer Imaging Archive (https://www.cancerimagingarchive.net/collection/cptac-luad, https://www.cancerimagingarchive.net/collection/cptac-lscc), whereas the CPTAC lung cancer bulk transcriptomics data is available from the GDC portal (https://portal.gdc.cancer.gov/projects/CPTAC-3). Whole slide imaging and clinical data from the SUNY Upstate cohort is currently not publicly available owing to restrictions of patient privacy.

## Code availability

The codes to implement HistoTME are publicly available at: https://github.com/spatkar94/HistoTME.git.
