# Author Correction: Predicting the tumor microenvironment composition and immunotherapy response in non-small cell lung cancer from digital histopathology images

**DOI:** 10.1038/s41698-024-00796-3

**Published:** 2025-01-09

**Authors:** Sushant Patkar, Alex Chen, Alina Basnet, Amber Bixby, Rahul Rajendran, Rachel Chernet, Susan Faso, Prashanth Ashok Kumar, Devashish Desai, Ola El-Zammar, Christopher Curtiss, Saverio J. Carello, Michel R. Nasr, Peter Choyke, Stephanie Harmon, Baris Turkbey, Tamara Jamaspishvili

**Affiliations:** 1 Artificial Intelligence Resource (AIR), National Cancer Institute, National Institutes of Health, Bethesda, MD USA; 2 Department of Hematology and Oncology, SUNY Upstate Medical University, Syracuse, NY USA; 3 Department of Pathology and Laboratory Medicine, SUNY Upstate Medical University, Syracuse, NY USA

**Keywords:** Cancer imaging, Cancer microenvironment, Non-small-cell lung cancer, Tumour biomarkers

Correction to: *npj Precision Oncology* 10.1038/s41698-024-00765-w, published online 19 December 2024

“In this article the author name Prashanth Ashok Kumar was incorrectly written as Prashant A. Kumar. The original article has been corrected.”

