# Supplementary material for: Predicting the tumor microenvironment composition and immunotherapy response in non-small cell lung cancer from digital histopathology images
Source: NPJ Precis Oncol. 2024 Dec 19;8:280. doi: 10.1038/s41698-024-00765-w (PMC11659524; doi:10.1038/s41698-024-00765-w)
Supplement: Supplementary file 1 — Supplementary Information [file 41698_2024_765_MOESM1_ESM.docx]

# Supplementary Figures

**Supplementary Figure 1:** **Overview of the study design**. To train HistoTME matched whole slide imaging and bulk RNA sequencing data from 865 patients from TCGA was utilized. To validate HistoTME matched whole slide imaging and bulk RNA sequencing data from 333 patients from CPTAC was utilized. For testing HistoTME predictions, matched whole slide H&E imaging and immunohistochemistry imaging data from surgical resection specimens of 82 patients from SUNY Upstate Medical University was utilized. For evaluating ICI efficacy surgical resection or needle biopsy specimens from 290 patients of the test cohort with matched clinical follow-up data following ICI treatment was utilized.

**Supplementary Figure 2:** **Scatter plots between the observed values derived from transcriptomics and HistoTME predicted values.** **(A)** Scatter plots between the observed values derived from transcriptomics and HistoTME predicted values is shown at the patient level on the CPTAC validation cohort (N=333). **(B)** Scatter plots between the cell type density, defined as the number of marker positive cells per mm^2^ from the immunohistochemistry (IHC) stain, and HistoTME predicted values is shown at the patient level on the external SUNY cohort (N=79). Cell type densities were quantified from whole slide immunohistochemistry images using QuPath v0.5.0 cell detection and classification algorithms and default parameters.

**Supplemental Figure 3**: **Pearson correlation between the observed values derived from transcriptomics and each model configuration’s predicted values.**  Pearson correlations are shown at the patient level on the external CPTAC validation cohort for antitumor, protumor, angiogenesis/stromal, and cancer/malignant cell-related signatures. Model configurations consisted of single-task and multi-task AB-MIL with CTransPath, RetCCL, or UNI as the feature extractor. Error bars represent the 95% confidence intervals. The mean, minimum, and maximum for each model across all TME signatures is shown in the table.

**Supplementary Figure 4: Bootstrapped Silhouette score analysis to determine the optimal number of clusters from K means clustering**. This analysis reveals K= 2 clusters maximize the average silhouette width. The input to the clustering algorithm were HistoTME-predicted expression of 30 TME signatures for TCGA+CPTAC-NSCLC patients

**Supplementary Figure 5: Break down of clinical characteristics of patients across HistoTME-predicted TME subtypes**. Stacked bar plots depict distribution of grade (panel A), histologic subtype (panel B), specimen type (panel C), stage (panel D), tissue site (panel E), PD-L1 scores (panel F). LUAD: Lung adenocarcinoma, LUSC: Lung squamous cell carcinoma. Smoking history: Current smoker, Former smoker and Never smoker (panel G). Patient age in years (panel H). Statistical significance between groups was determined using the Chi-squared test.

**Supplementary Figure 6: Distribution of pairwise Pearson correlations between attention maps**. Pairwise Pearson correlations between attention maps of antitumor, protumor, angiogenesis/stroma, and malignant cell signatures are calculated on the SUNY cohort (652 patients, 1329 slides). Each data point in the distribution represents a single whole slide image.


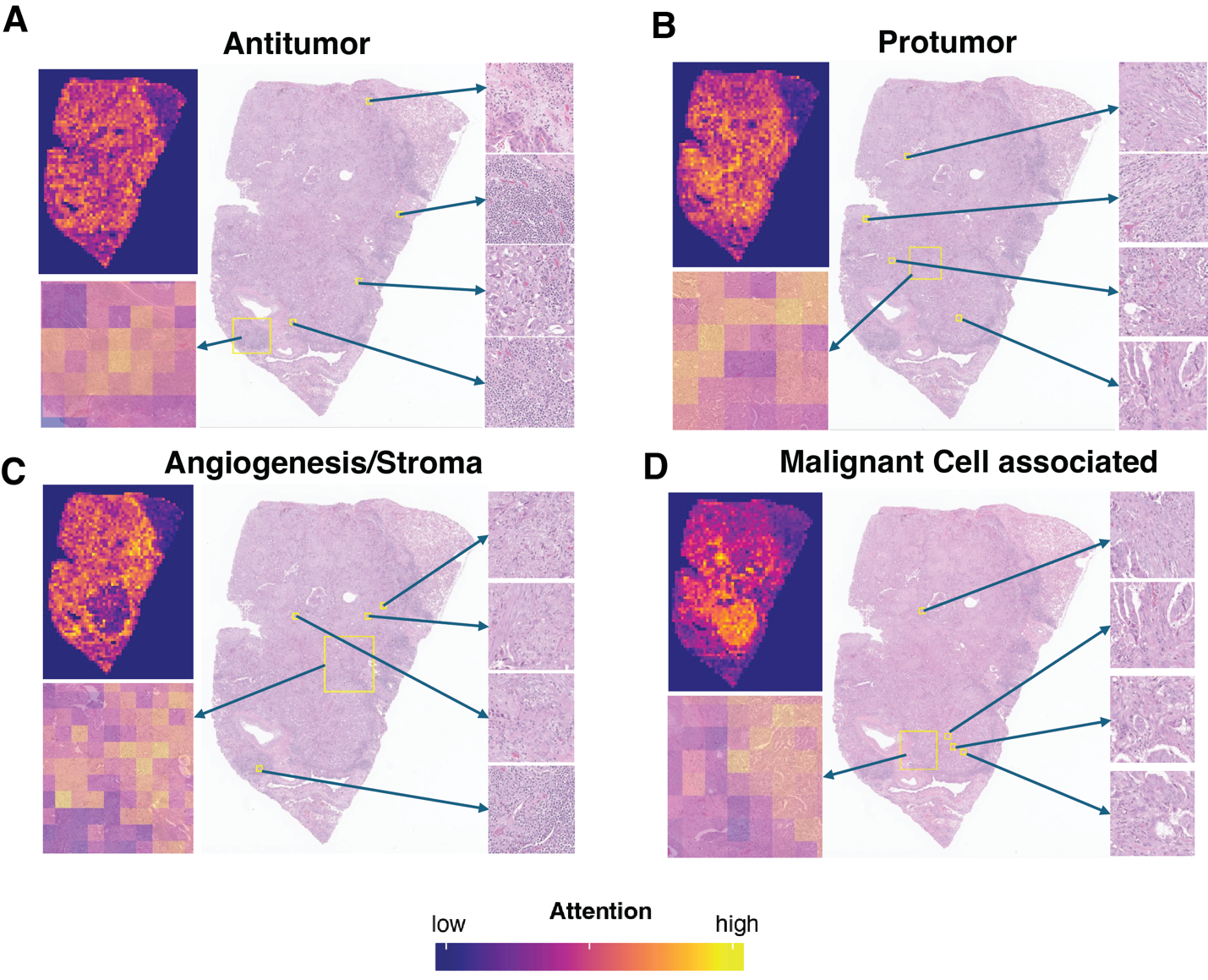


**Supplementary Figure 7: HistoTME-generated attention maps for a case predicted to have an Immune Inflamed TME.** **(A)** attention maps highlighting regions of interest corresponding to antitumor immune signatures **(B)** attention maps highlighting regions of interest corresponding to protumor immune signatures. **(C)** attention maps highlighting regions of interest corresponding to angiogenesis/stroma-associated signatures **(D)** attention maps highlighting regions of interest corresponding to malignant cell-associated signatures. Each tile highlighted corresponds to 256µm in size.


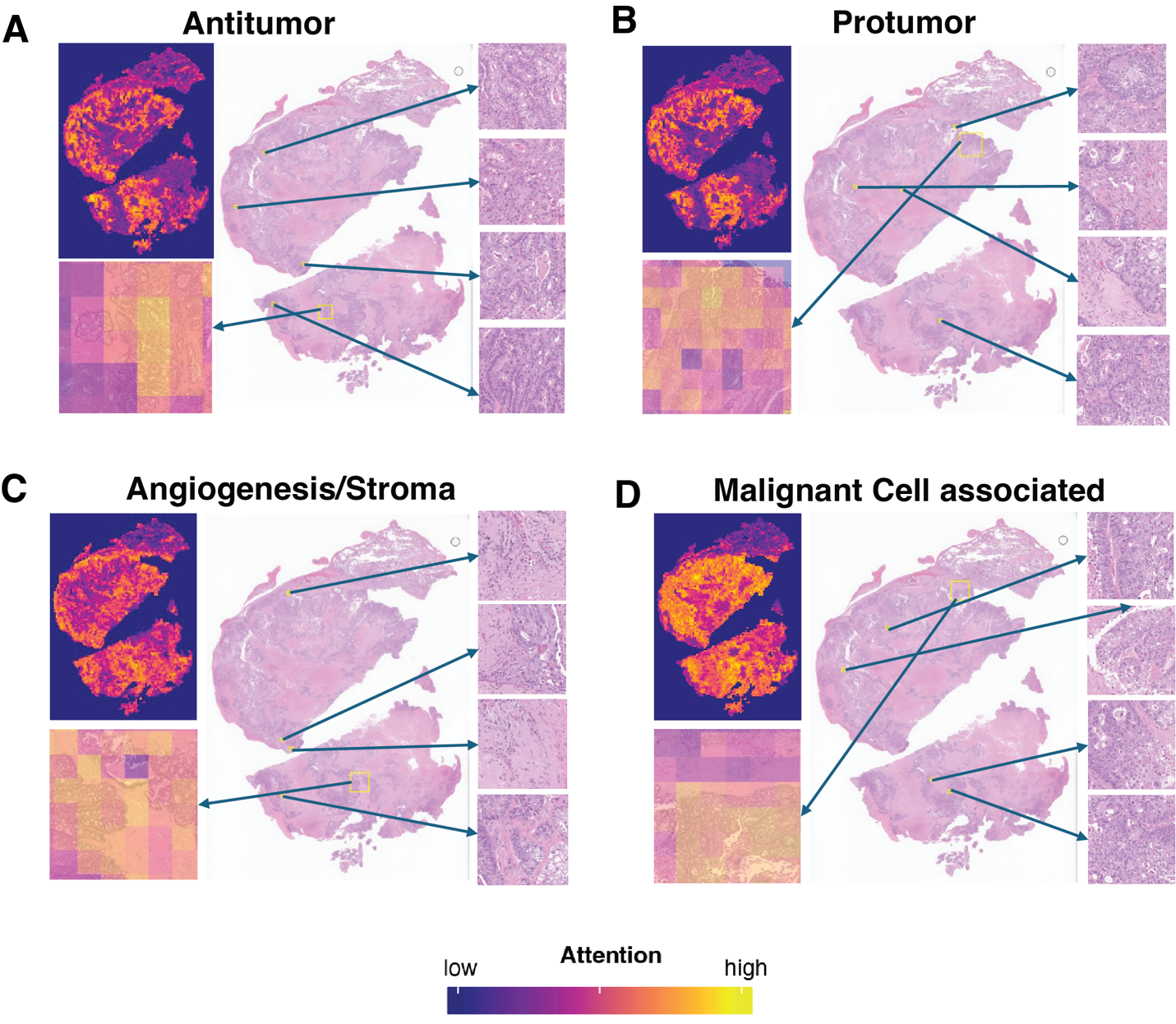


**Supplementary Figure 8: HistoTME-generated attention maps for a case predicted to have an Immune Desert TME.** **(A)** attention maps highlighting regions of interest corresponding to antitumor immune signatures **(B)** attention maps highlighting regions of interest corresponding to protumor immune signatures. **(C)** attention maps highlighting regions of interest corresponding to angiogenesis/stroma-associated signatures **(D)** attention maps highlighting regions of interest corresponding to malignant cell-associated signatures. Each tile highlighted corresponds to 256µm in size.


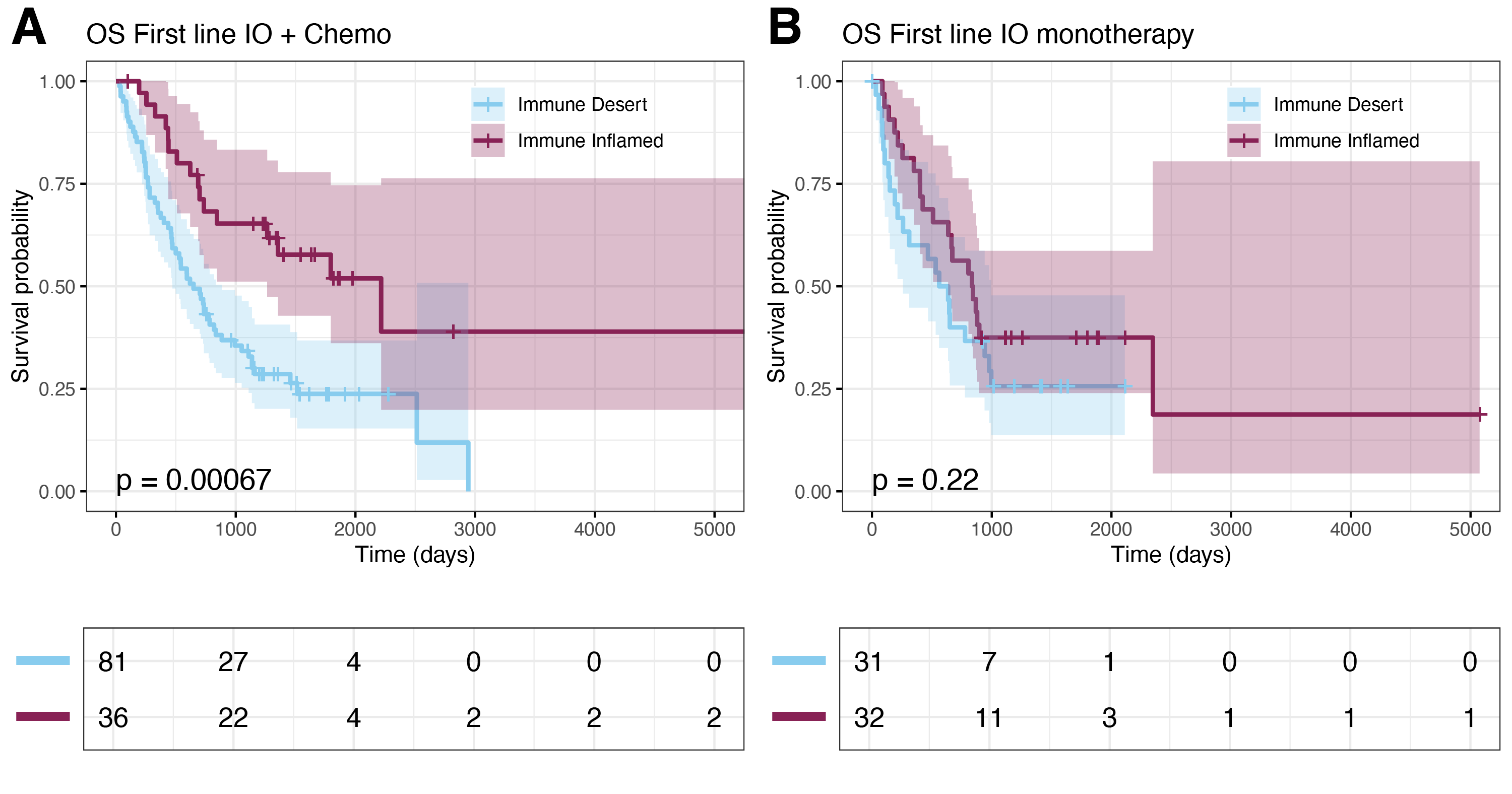


**Supplementary Figure 9:** **Association between HistoTME-based TME classification and overall survival outcomes of SUNY NSCLC patients treated with first-line ICI as combination therapy (first-line IO + Chemo) and monotherapy (IO monotherapy).** **(A)** Kaplan Meier plot depicting overall survival−defined as time from date of diagnosis to date of death −of patients that received first-line IO + chemo **(B)** Kaplan Meier plot depicting overall survival of SUNY patients that received first-line IO monotherapy. Significance of survival differences between distinct subgroups of patients was determined by the log-rank test.


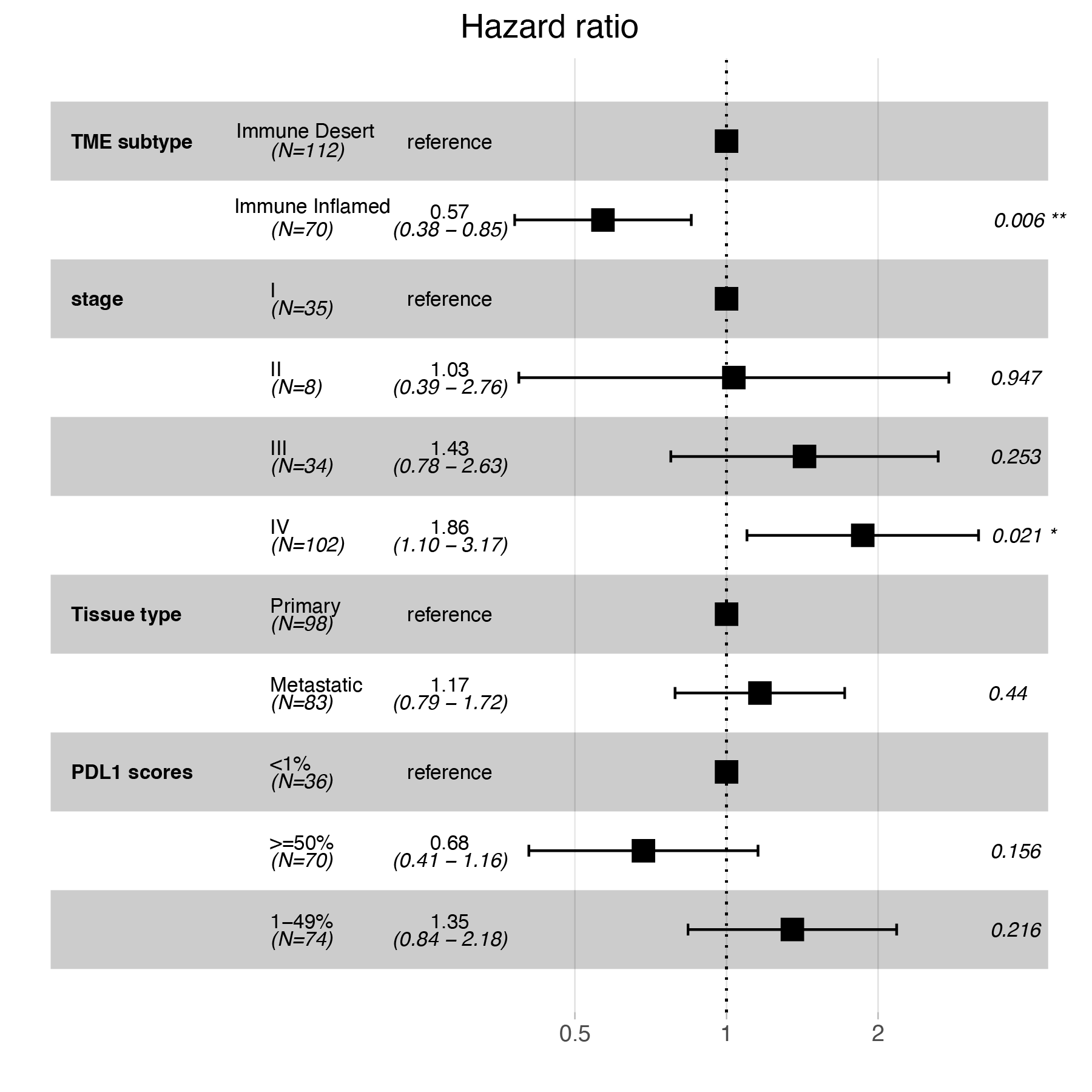


**Supplementary Figure 10: Multivariate Cox Proportional Hazards Analysis of Prognostic Factors in NSCLC Patients with first-line ICI therapy.** This forest plot depicts the hazard ratios for NSCLC patients who received immune checkpoint inhibitor (ICI) as first line therapy. The analysis was conducted using a multi-variable Cox proportional hazards model accounting for confounding factors. Significant associations are indicated by asterisks (*)


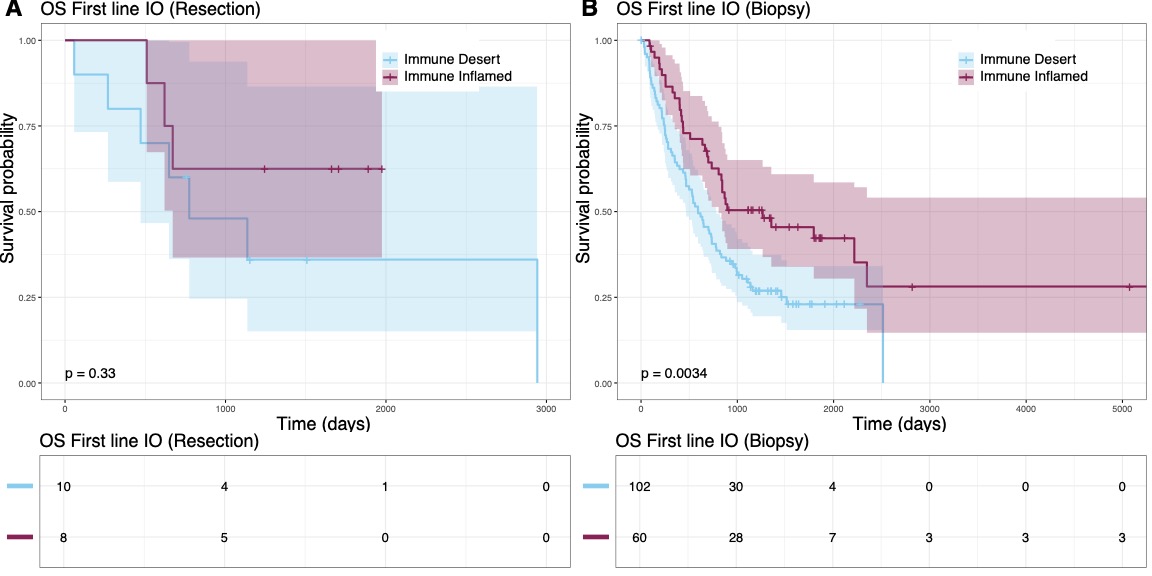


**Supplementary Figure 11: Specimen source subgroup analysis of NSCLC patients treated with First line ICI therapy.** Kaplan Meier plots depicting the overall survival trends for NSCLC patients who received immune checkpoint inhibitor (ICI) as first line therapy. (A) depicts TME subtypes of patients based on analysis of surgical resection slides whereas (B) depicts TME subtypes of patients based on analysis of needle biopsy slides. Statistical significance of differences in survival curves was determined using the log-rank test.


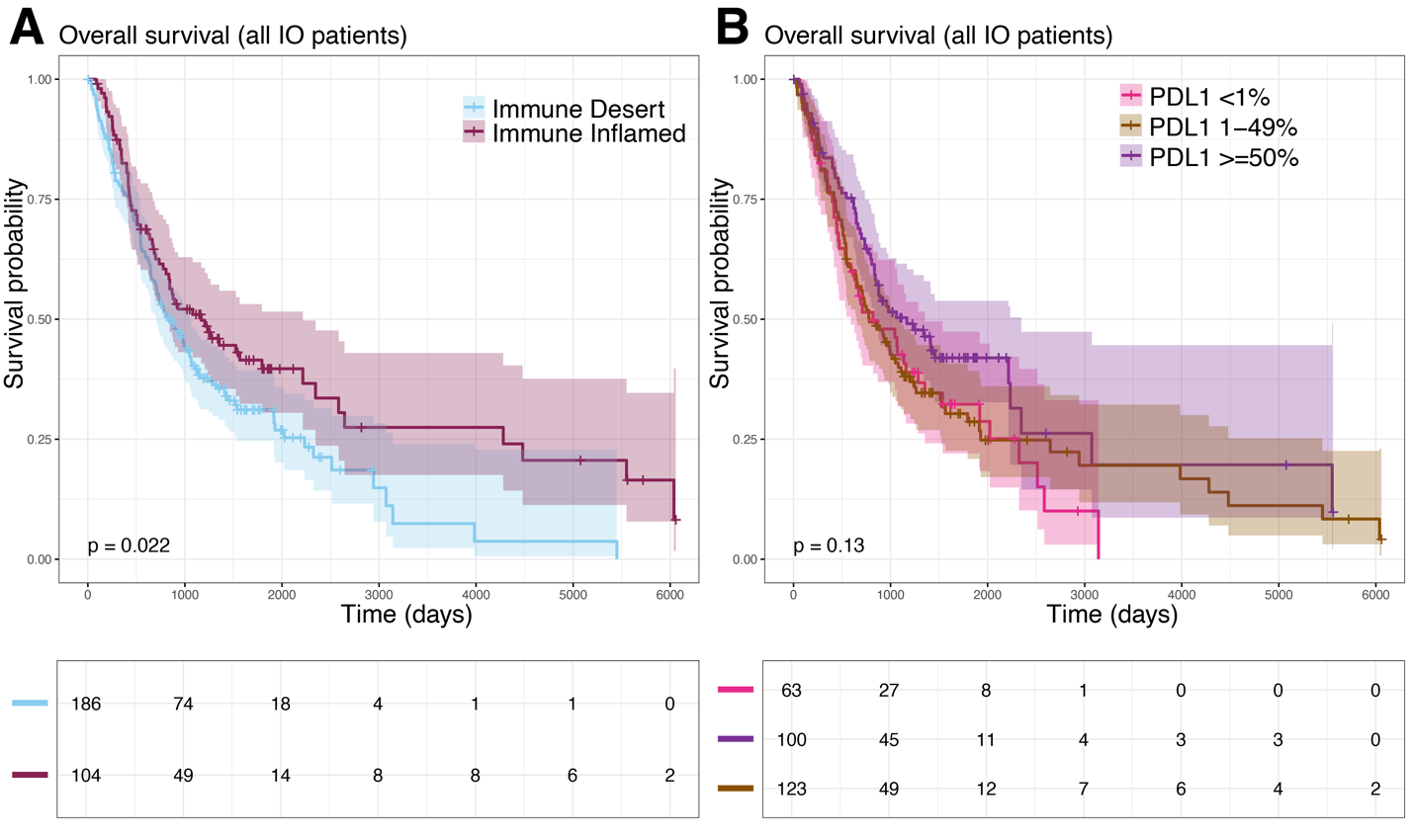


**Supplementary Figure 12: Overall survival (OS) of all immunotherapy (IO)-treated patients** **(A)** stratified by Immune Inflamed and Immune Desert TME subtype **(B)** stratified by PD-L1 expression. Statistical significance between groups was estimated using the log-rank test.


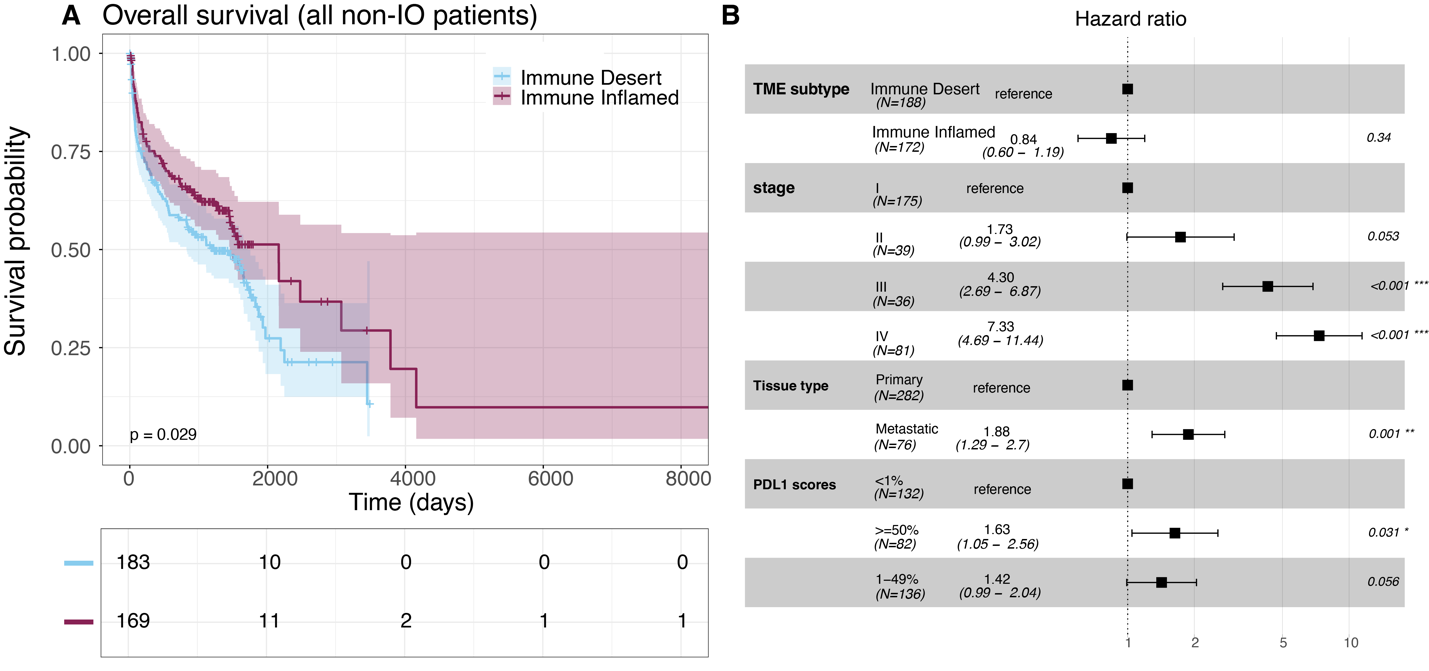


**Supplementary Figure 13: Survival Analysis of NSCLC Patients not treated with ICI therapy.** (A) Kaplan-Meier survival plot depicting overall survival rates of immune inflamed and immune desert patients that did not receive immunotherapy. (B) forest plot depicting the hazard ratios for NSCLC patients who did not receive immune checkpoint inhibitor (ICI) therapy. The analysis was conducted using a multi-variable Cox proportional hazards model, accounting for confounding factors. Significant associations are indicated by asterisks (*)


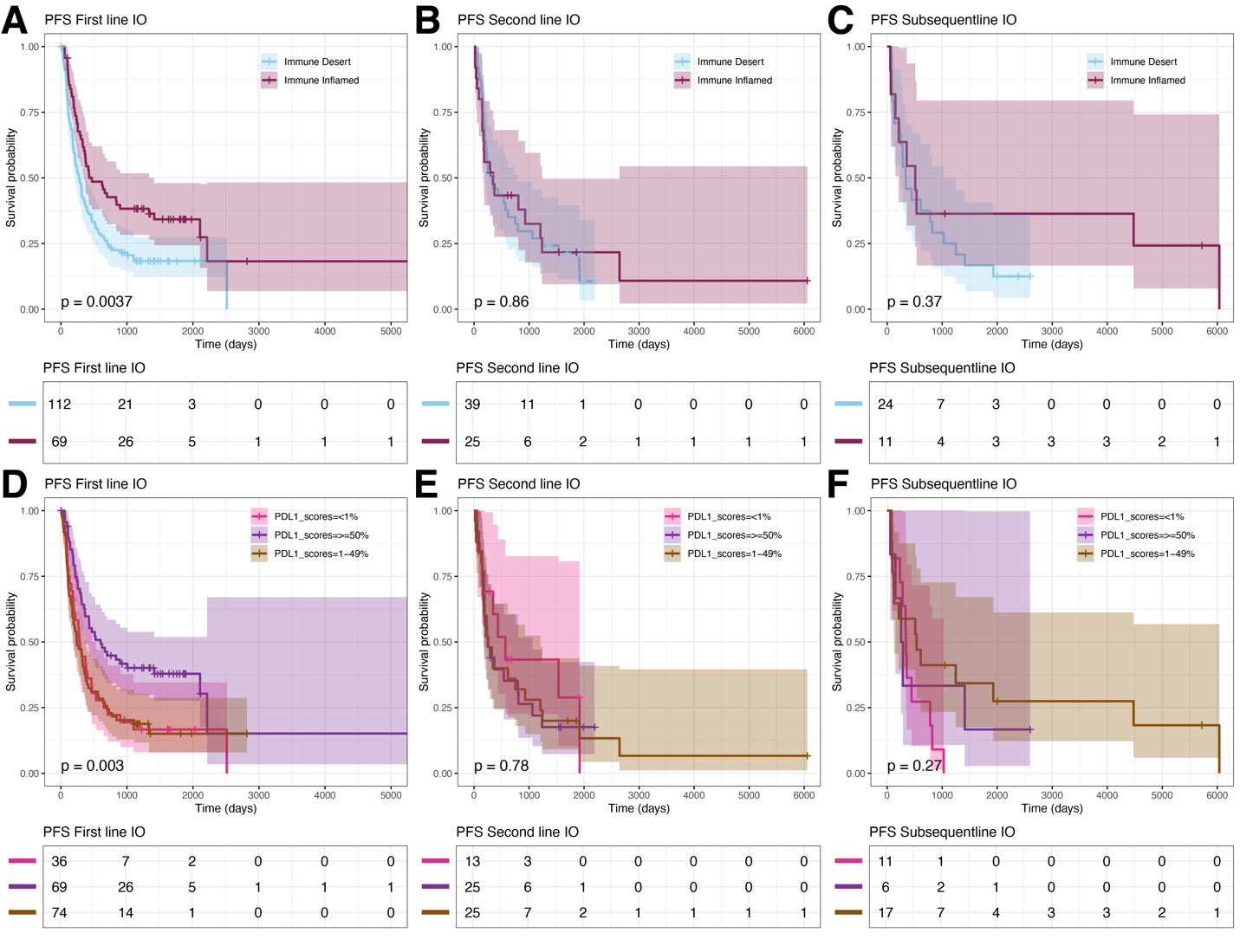


**Supplementary Figure 14: Progression free survival (PFS) of immunotherapy (IO)-treated patients (A-C)** Progression-free survival (PFS) of **(A)** first-line immunotherapy (IO)-treated, **(B)** second-line IO-treated, or **(C)** subsequent-line IO-treated patients in the SUNY Upstate cohort stratified by TME subtype. **(D-F)** Progression-free survival (PFS) of **(D)** first-line immunotherapy (IO)-treated, **(E)** second-line IO-treated, or **(F)** subsequent-line IO-treated patients in the SUNY Upstate cohort stratified by PD-L1 expression. Statistical significance between groups was estimated using the log-rank test.


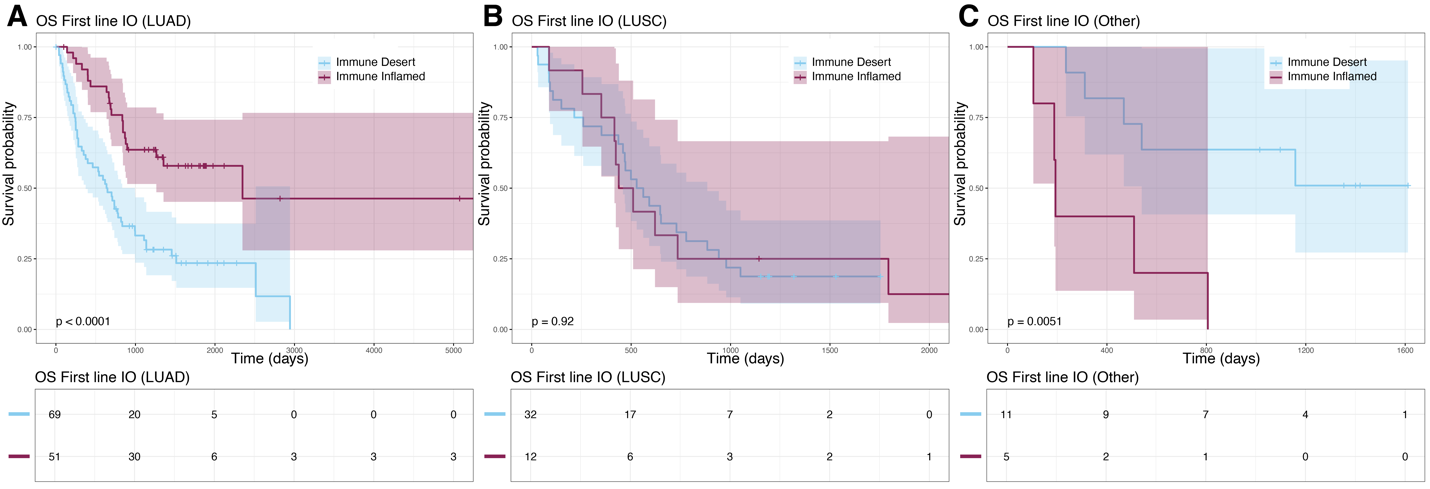


**Supplementary Figure 15: Histologic subtype-based subgroup analysis of NSCLC patients treated with First line ICI therapy.** Kaplan Meier plots depicting the overall survival trends for NSCLC patients who received immune checkpoint inhibitor (ICI) as first line therapy. (A) depicts TME subtypes of lung adenocarcinoma patients whereas (B) depicts TME subtypes of squamous cell carcinoma patients and (C) depicts TME subtypes of other (neuroendocrine/pleomorphic) patients. Statistical significance of differences in survival curves was determined using the log-rank test.


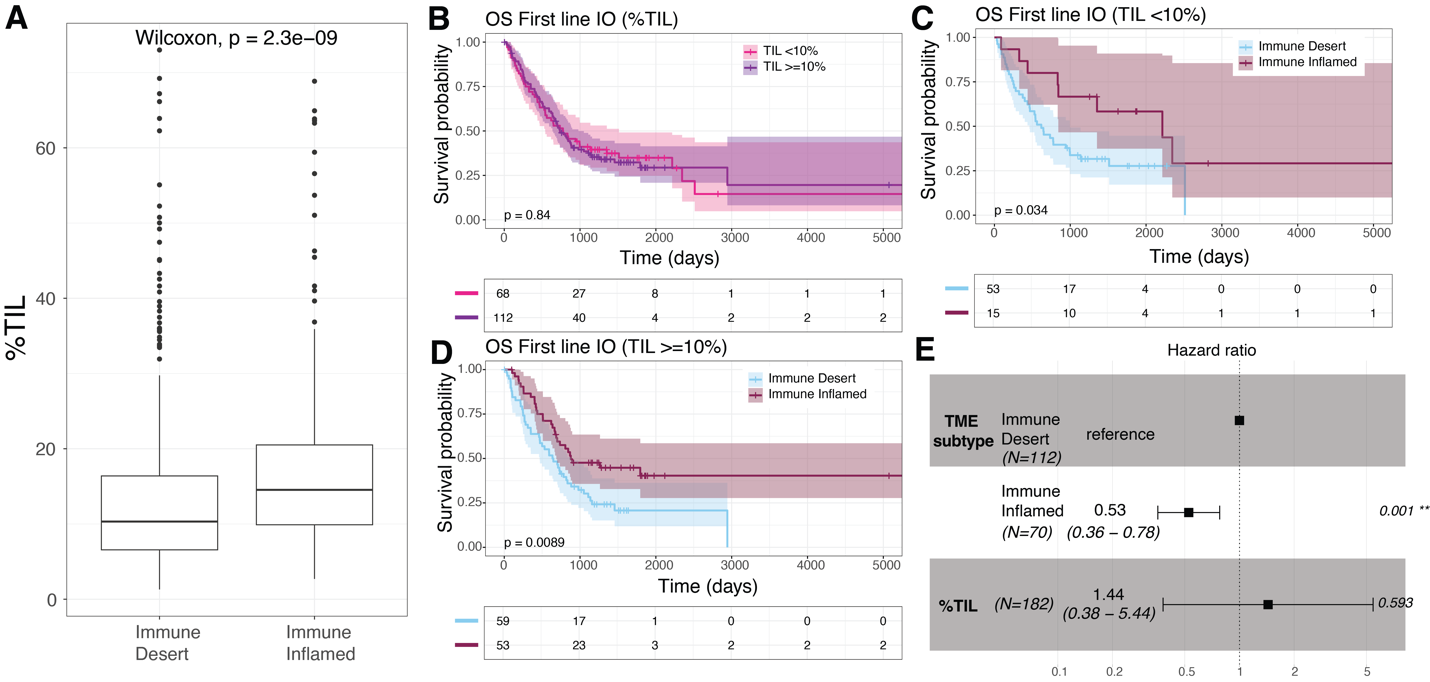


**Supplementary Figure 16: Benchmarking prognostic value of HistoTME-predicted TME subtypes against %TIL counts**. (A) Distribution of %TIL counts estimated using automated cell segmentation and classification tool CellVIT (PMID: 38507894). Statistical significance between groups was evaluated using Wilcox rank sum test. (B) Kaplan Meier plot depicting overall survival differences of first line ICI-treated patients based on %TILs counts derived from CellVIT. (C) & (D) Kaplan Meier plot depicting overall survival differences of patients classified as Inflamed or Desert within each TIL subgroup. Statistical significance between Inflamed and Desert groups was determined using the log-rank test. (E) Cox proportional hazards ratios estimated for HistoTME-derived TME subtypes and CellVIT-derived TIL%. Significant associations are indicated by asterisks (*)

**Supplementary Figure 17: Overview of Random Forest based feature selection process.** Random forest feature selection that maximized 5-fold cross-validation AUROC when XGBoost was trained to predict ICI response using **(A)** pairwise engineered TME signature interactions and **(B)** TME signatures alone. The number of features where AUROC was maximized was used.

# Supplementary Figure 18: Progression-free survival (PFS) of immunotherapy (IO)-treated patients in the test cohort. (A) first-line immunotherapy (IO)-treated, (B) second-line IO-treated, or (C) subsequent-line IO-treated patients in the SUNY held-out test set (N=69) predicted to be responders or non-responders. Statistical significance between groups was estimated using the log-rank test.

# Supplementary Tables

**Supplementary Table 1** Summary of the clinical demographics of the SUNY Upstate NSCLC cohort. A total of 652 patients with whole slide imaging and/or clinical follow-up data were analyzed in this study. PD-L1 manual scoring was performed by expert pathologists using an FDA-approved assay and scoring guidelines at LabCorp. In an event where multiple biopsies were taken from a single patient, the overall PD-L1 score assigned to that patient was maximum of PD-L1 scores assigned to each biopsy. The full clinical table is accessible from the GitHub repository.

**Supplementary Table 2** Overview of reagents used for serial immunohistochemical staining of surgical resection specimens from the SUNY Upstate NSCLC cohort.
